# Risk of Long COVID in hospitalized individuals treated with remdesivir for acute COVID-19

**DOI:** 10.1038/s41598-025-06052-3

**Published:** 2025-07-28

**Authors:** Mark Berry, Amanda M. Kong, Roger Paredes, Julie Paone, Rohan Shah, Rebecca Taylor, Essy Mozaffari, Rikisha Gupta, Robert L. Gottlieb, Lourdes Mateu, Mazin Abdelghany, Jason D. Goldman, Anand P. Chokkalingam

**Affiliations:** 1 Gilead Sciences, Inc., Foster City, CA USA; 2 Aetion, Inc., 5 Penn Plaza, New York, NY USA; 3 IrsiCaixa, Ctra. de Canyet, Barcelona, Spain; 4 Department of Infectious Diseases, Hospital Universitari Germans Trias i Pujol, Barcelona, Spain; 5 Baylor University Medical Center, Dallas, TX USA; 6 Baylor Scott & White Health, Dallas, TX USA; 7 Texas A&M Health Science Center, Dallas, TX USA; 8 Burnett School of Medicine at TCU, Fort Worth, TX USA; 9 Swedish Center for Research and Innovation, Providence Swedish Medical Center, Seattle, WA USA; 10 Division of Infectious Diseases, Providence Swedish Medical Center, 1124 Columbia St. #600, Seattle, WA 98104 USA; 11 Division of Allergy and Infectious Diseases, University of Washington, Seattle, WA USA; 12 School of Public Health, University of California, Berkeley, CA USA

**Keywords:** COVID-19, Long COVID, Antiviral therapy, Remdesivir, Immunocompromising conditions, Viral infection, Drug development

## Abstract

Long COVID comprises a multisystem syndrome occurring after COVID-19. This retrospective cohort study investigated whether remdesivir given during acute COVID-19 is associated with reduced incidence of Long COVID, including in immunocompromised subgroups. The HealthVerity database of hospital chargemaster data linked to closed claims was queried for patients aged ≥ 12 years hospitalized for ≥ 2 days with COVID-19 between May 1, 2020, and September 30, 2021. Relative risk between remdesivir-exposed and unexposed patients was calculated for 16 individual Long COVID outcomes and a composite of any Long COVID outcome, occurring 90–270 days after hospital admission. Subgroup analyses occurred in immunocompromised patients. Regression models accounted for censoring, competing risks, and treatment assignment weights; statistical inferences were adjusted for multiple comparisons. Among 3,661,303 hospitalized patients, 52,006 with COVID-19 were included; 20,246 (38.9%) were immunocompromised. In the overall and immunocompromised populations, respectively, 33.0% and 29.5% received remdesivir; the composite of ≥ 1 Long COVID outcome occurred in 55.5% and 62.9%. Patients administered remdesivir experienced lower risk of any Long COVID outcome (risk ratio, 0.96; 95% CI 0.94–0.97; adjusted *P* < 0.001). Risk for several individual Long COVID outcomes was lower in those receiving remdesivir in the overall and immunocompromised populations. In conclusion, exposure to remdesivir was associated with a lower risk of Long COVID.

## Introduction

Long COVID is a persistent, multisystem syndrome occurring after SARS-CoV-2 infection that may last years and can result in significant disability^1,2^. Long-COVID–associated symptoms are highly variable and include malaise, fatigue, altered smell and taste, breathlessness, and cognitive impairment^1,3,4^. Recently, the National Academies proposed a standardized case definition for Long COVID, though other definitions exist^5–7^. The World Health Organization (WHO) has proposed a clinical case definition as the continuation or development of new symptoms ≥ 3 months after COVID-19 onset that lasts for ≥ 2 months with no alternative explanation^6^. Long COVID is estimated to occur in ≥ 10% of COVID-19 survivors^8^. In individuals who were hospitalized for acute COVID-19, the risk of Long COVID was nearly 3 times greater, with a longer duration of symptoms^8,9^.

Relative to the general population, individuals with immunocompromising conditions are at increased risk of progression to severe COVID-19^10–12^. Although progression to severe COVID-19 is a known risk factor for Long COVID^8^, its incidence in this population is unknown^1,3,12^. Thus, individuals with immunocompromising conditions represent a key population for treatment during acute COVID-19^10^.

A key hypothesis of Long COVID pathophysiology is the establishment of a viral reservoir^1^. SARS-CoV-2 viremia during acute COVID-19 predicts later development of Long COVID^13^. Furthermore, circulating SARS-CoV-2 spike antigen can persist up to 12 months after acute infection and is associated with Long COVID^14^. Studies have shown that ritonavir-boosted nirmatrelvir (labeled for outpatient use) reduces the risk of Long COVID in nonhospitalized patients^15,16^. Data for remdesivir are mixed, although observational studies have been limited by small sample sizes and relatively low acuity (low rates of intensive care unit admission or supplemental oxygen use)^17,18^. Whether antivirals given during hospitalization for acute COVID-19 decrease the incidence of Long COVID remains a key knowledge gap.

Remdesivir has demonstrated efficacy in the treatment of COVID-19 across variant eras^19–24^. Remdesivir is indicated for use in nonhospitalized individuals at high risk for disease progression and hospitalized individuals not requiring mechanical ventilation^25,26^. Observational studies have suggested favorable clinical outcomes in individuals with immunocompromising conditions treated with remdesivir^23,27,28^. However, the impact of remdesivir on Long COVID is unknown, including in individuals with immunocompromising conditions. Here, the association of remdesivir use during acute COVID-19 and later development of Long COVID–related conditions was assessed in patients hospitalized with COVID-19 from the overall population and a subgroup with immunocompromising conditions.

## Results

**Fig. 1:**
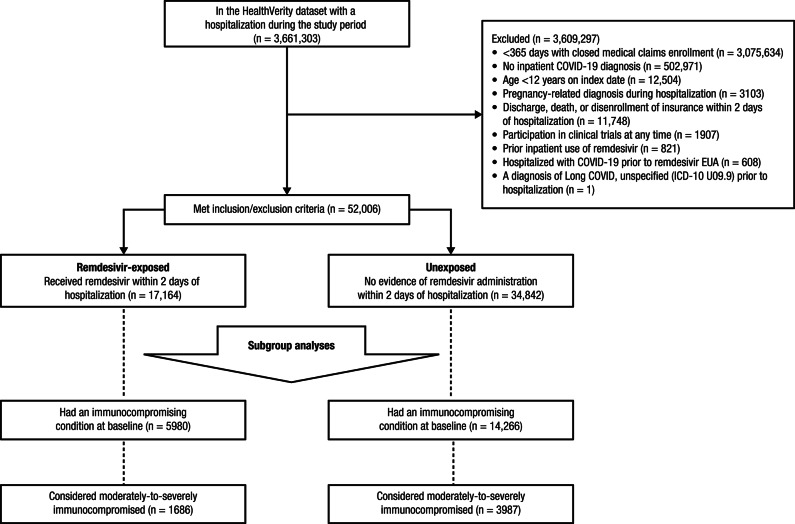
CONSORT diagram. The enrollment and inclusion or exclusion of patients from the HealthVerity Real-time Insights and Evidence database. Abbreviations: EUA, emergency use authorization; ICD-10, *International Classification of Diseases, Tenth Revision*.

A total of 3,661,303 patients had ≥ 1 hospitalization between May 1, 2020, and September 30, 2021, in the chargemaster data (Fig. 1). Overall, 52,006 patients hospitalized with COVID-19 met inclusion criteria. The mean age was 59 years, 26,522 (51.0%) were female, 17,164 (33.0%) were remdesivir-exposed, and 34,842 (67.0%) were unexposed (Table 1). Additionally, 20,246/52,006 (38.9%) patients had an immunocompromising condition during the baseline period (remdesivir-exposed: 5980 [29.5%]; unexposed: 14,266 [70.5%]) while 5673/52,006 (10.9%) were considered moderately-to-severely immunocompromised (remdesivir-exposed:1686 [29.7%]; unexposed: 3987 [70.3%]; Supplementary Table 1). Nearly all (16,297/17,164 [94.9%]) patients who were remdesivir-exposed received corticosteroid treatment versus 17,552/34,842 (50.4%) of those unexposed.

**Table 1:** Demographic and Baseline Characteristics.

Characteristic	Total (N = 52,006)	Remdesivir-exposed (n = 17,164)	Unexposed (n = 34,842)	*SMD*
Sex, n (%)				0.06
Male	25,484 (49.0)	8727 (50.8)	16,757 (48.1)	
Female	26,522 (51.0)	8437 (49.2)	18,085 (51.9)	
Age, years, mean (SD)	59 (18.3)	57 (16.4)	60 (19.1)	0.13
Maximal level of oxygen support on Day 0 or 1, n (%)				0.23
Room/ambient air	33,810 (65.0)	10,006 (58.3)	23,804 (68.3)	
Low-flow oxygen	10,458 (20.1)	4189 (24.4)	6269 (18.0)	
High-flow oxygen/noninvasive ventilation	5265 (10.1)	2209 (12.9)	3056 (8.8)	
ECMO/invasive mechanical ventilation	2473 (4.8)	760 (4.4)	1713 (4.9)	
ICU admission on Day 0 or 1, n (%)	25,221 (48.5)	8608 (50.2)	16,613 (47.7)	0.05
Concomitant medications received on Day 0 or 1, n (%)				
Corticosteroids	33,849 (65.1)	16,297 (94.9)	17,552 (50.4)	1.16
Immunomodulators	1860 (3.6)	1390 (8.1)	470 (1.3)	0.32
Convalescent plasma	2730 (5.2)	2070 (12.1)	660 (1.9)	0.41
Anticoagulants	13,949 (26.8)	3161 (18.4)	10,788 (31.0)	0.29
Immunocompromised* during baseline period,^†^ n (%)	20,246 (38.9)	5980 (34.8)	14,266 (40.9)	0.13
Moderately-to-severely immunocompromised during baseline period,^†^ n (%)	5673 (10.9)	1686 (9.8)	3987 (11.4)	0.05

ECMO, extracorporeal membrane oxygenation; ICU, intensive care unit; NIH, National Institutes of Health; SMD, standardized mean difference.

*Immunocompromised by modified NIH definition (Table 1) ^30^. ^†^This variable was not accounted for in propensity score weighting.

The composite outcome of coding of ≥ 1 Long COVID–associated outcome or International Classification of Diseases, Tenth Revision (ICD-10) code U09.9 occurred in 28,845/52,006 (55.5%) patients in the overall population, 12,736/20,246 (62.9%) in the immunocompromised population, and 3684/5673 (64.9%) in the moderately-to-severely immunocompromised population. The numbers of patients who were remdesivir-exposed or unexposed and who had evidence of inpatient death, were censored, completed 270 days of follow-up, or had an outcome (eg, any Long COVID outcome) are presented in Supplementary Table 2. Among patients who had ≥ 90 days of follow-up in the overall population (n = 45,587), the immunocompromised population (n = 17,480), and the moderately-to-severely immunocompromised population (n = 4889), the rate of Long COVID outcomes was 283, 397, and 436 per 100 person-years, respectively (Table 2). The most common Long COVID outcomes were neuropsychiatric features, breathlessness, fatigue, and joint pain; the least common outcomes were taste disturbance/dysgeusia/ageusia, smell disturbance/anosmia, and dysautonomia. Standardized mean differences (SMDs) after weighting across all outcomes are shown in Supplementary Table 3. The SMDs for corticosteroid use tended to be higher (~ 0.2), as was the SMD for being hospitalized in May 2020.

**Table 2:** Rate of Long COVID–associated outcomes after hospitalization with COVID-19 in the Overall Population and in Immunocompromised Subgroups With ≥ 90 Days of Follow-up.

	Immunocompromised patients (n = 17,480)		Moderately-to-severely immunocompromised* patients (n = 4889)		Overall population (N = 45,587)	
Outcome	n	Rate per 100 person-years (95% CI)^†^	n	Rate per 100 person-years (95% CI)^†^	n	Rate per 100 person-years (95% CI)^†^
Any potential Long COVID outcome	12,736	397 (389.7–403.5)	3684	436 (421.6–449.8)	28,845	283 (277.6–284.0)
Neuropsychiatric features	7721	150 (146.6–153.2)	2337	170 (162.8–176.6)	16,877	115 (112.2–115.6)
Dyspnea/breathlessness	4208	63 (61.6–65.4)	1509	87 (82.9–91.7)	8501	47 (46.0–48.0)
Fatigue	4162	63 (60.7–64.5)	1296	72 (67.9–75.7)	8233	45 (44.3–46.3)
Joint pain/arthralgia	3931	59 (56.7–60.4)	1151	62 (58.8–66.0)	7804	43 (41.7–43.5)
Cognitive dysfunction	3430	51 (49.4–52.8)	860	44 (41.5–46.7)	7230	40 (38.2–40.0)
Chest pain	3388	49 (47.4–50.7)	1191	65 (60.9–68.2)	6722	36 (35.1–36.8)
Cerebrovascular disease	2435	34 (32.9–35.6)	625	31 (29.0–34.0)	4947	26 (25.2–26.7)
Thromboembolic disease	1765	24 (23.0–25.3)	580	29 (26.5–31.2)	3303	17 (16.3–17.5)
Cough	1753	24 (22.6–24.9)	632	31 (29.0–33.9)	3553	18 (17.6–18.7)
Ischemic heart disease	1450	19 (18.4–20.4)	449	22 (19.7–23.8)	2753	14 (11.5–12.5)
Diarrhea	1172	15 (14.6–16.4)	422	20 (18.4–22.3)	2021	10 (9.6–10.5)
Headache	705	9 (8.5–9.8)	246	12 (10.1–13.0)	1396	7 (6.5–7.3)
Muscle pain/myalgia	683	9 (8.2–9.6)	234	11 (9.7–12.5)	1234	6 (5.8–6.4)
Dysautonomia	30	0.4 (0.2–0.5)	15	0.7 (0.3–1.0)	50	0.2 (0.2–0.3)
Taste disturbance/dysgeusia/ageusia	18	0.2 (0.1–0.3)	4	0.2 (0.0–0.4)	40	0.2 (0.1–0.3)
Smell disturbance/anosmia	16	0.2 (0.1–0.3)	7	0.3 (0.1–0.6)	46	0.2 (0.2–0.3)

NIH, National Institutes of Health. *Immunocompromised by modified NIH definition^30^. ^†^Person-time was calculated as the number of days between Day 90 following hospitalization and the earliest of the following events: occurrence of the outcome, inpatient death, disenrollment, data cutoff (April 30, 2022), or Day 270 after admission.

**Fig. 2:**
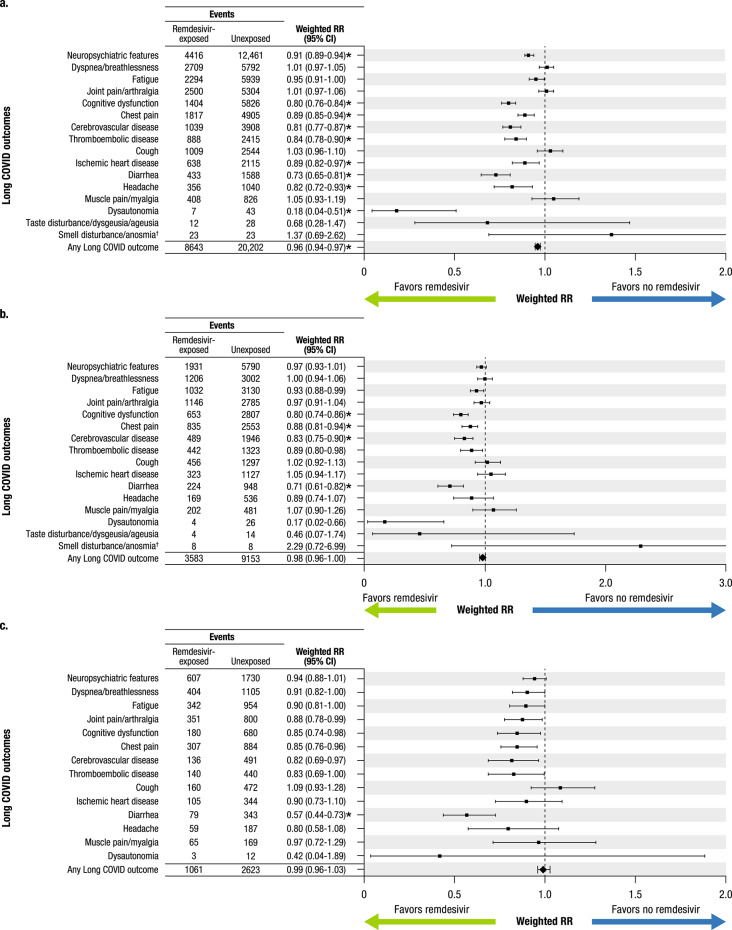
Association of remdesivir with Long COVID outcomes after hospitalization with COVID-19*.* The weighted risk of Long COVID outcomes associated with remdesivir administration compared with no remdesivir administration is shown in descending order of total events for (**a**) The overall study population, (**b**) Individuals with immunocompromising conditions, and (**c**) Individuals who are moderately-to-severely immunocompromised. For the moderately-to-severely immunocompromised subgroup, there were too few events to assess taste disturbance/dysgeusia/ageusia and smell disturbance/anosmia, and these are not shown. Relative risk was calculated with weighting by propensity score, competing risk, and disenrollment. Abbreviations: RR, risk ratio. *Statistically significant outcomes after adjusting statistical inference for multiple comparisons testing using the Holm-Bonferroni method (total α = 0.05; m = 17, where m is the number of hypotheses). ^†^Error bars for smell disturbance/anosmia extend beyond the shown scale.

Exposure to remdesivir was associated with a lower risk for the composite primary outcome of any Long COVID outcome (risk ratio, 0.96; 95% CI 0.94–0.97; *P* < 0.001) and for the individual outcomes of neuropsychiatric features, cognitive dysfunction, chest pain, cerebrovascular disease, thromboembolic disease, ischemic heart disease, diarrhea, headache, and dysautonomia after weighting and correction for multiple testing (Fig. 2A). Remdesivir was associated with a reduced risk of cognitive dysfunction, chest pain, cerebrovascular disease, and diarrhea among patients with immunocompromising conditions (Fig. 2B) and with a reduced risk of diarrhea in patients who were considered moderately-to-severely immunocompromised (Fig. 2C). Results for all other outcomes were nonsignificant, and none indicated increased risk associated with remdesivir.

There were 3256/34,842 (9.3%) patients in the unexposed group with evidence of remdesivir use after Day 2. In an as-treated sensitivity analysis where unexposed patients were censored on remdesivir use, remdesivir was associated with a lower risk of the composite outcome of any Long COVID outcome and for the individual Long COVID outcomes of neuropsychiatric features, cognitive dysfunction, chest pain, cerebrovascular disease, thromboembolic disease, ischemic heart disease, diarrhea, headache, and dysautonomia (Supplementary Fig. 1).

## Discussion

This study assessed the risk of developing Long COVID–associated outcomes following hospitalization with COVID-19 in a large population of US patients. After weighting and adjusting for multiple hypothesis testing, remdesivir administration was associated with a modestly reduced risk of several Long COVID outcomes in the overall study population and in the subsets of patients with immunocompromising conditions.

Remdesivir use was associated with a reduction in risk for a greater number of Long COVID outcomes in the overall population relative to the subpopulations of patients with immunocompromising conditions; this finding is of unclear significance. This may have been due to smaller sample sizes and reduced power in these subgroups. Additionally, though patients with immunocompromising conditions were identified using a previously published definition^29^, this subgroup is inherently heterogeneous. Moderately-to-severely immunocompromising conditions and multimorbidity are among the greatest risk factors for severe COVID-19, possibly predisposing these populations to worse outcomes^10–12,30^. Future studies should consider whether a longer course of remdesivir in immunocompromised individuals might reduce the risk of Long COVID in this population.

Compared with standard of care, remdesivir improves survival in hospitalized patients at high risk of progression to severe COVID-19^22,31,32^. At the time of the study, remdesivir was the only antiviral to receive emergency use authorization (EUA) in the United States and eventual approval for use in hospitalized patients; however, most patients did not receive remdesivir. The importance of early initiation of remdesivir is underlined in clinical guidelines^25,26^. Further studies of remdesivir’s effect on Long COVID in the outpatient setting are needed. Emerging evidence from real-world studies support a benefit of reduced mortality with remdesivir treatment, both in the overall population^33–35^ and in patients with immunocompromising conditions, irrespective of baseline supplemental oxygen requirements and across dominant variant periods^23^. Compared with earlier studies assessing remdesivir’s effect on the risk of Long COVID^17,18^, this study possessed a much larger sample size, including patients requiring intensive care unit and invasive mechanical ventilation/extracorporeal membrane oxygenation. This study was limited exclusively to patients indexed in an inpatient setting; thus, it is not possible to directly compare risk reductions in Long COVID with those seen for ritonavir-boosted nirmatrelvir^15,16^, which were assessed in nonhospitalized patients. These findings build on earlier studies by providing evidence that receipt of an antiviral during hospitalization with acute COVID-19 is associated with reduced risk of Long COVID.

This analysis was performed using a large dataset of hospitalized US patients, including many with immunocompromising conditions, and used weighting to ensure balance between remdesivir and comparator populations. The start of the 90-day posthospitalization observation period used here is consistent with that used in the WHO definition of Long COVID^6^. This study occurred when different SARS-CoV-2 variants predominated, up to and including the Delta variant. Further research should assess remdesivir’s impact on Long COVID outcomes in the periods of Omicron and currently circulating variants. Finally, this analysis utilized the Holm-Bonferroni method to lower the probability of a type 1 error, finding an association between remdesivir and an outcome spuriously due to the large number of outcome models. Consequently, other associations may not have been deemed significant (type 2 error).

This study had several limitations. The data analyzed in this study were collected between May 1, 2020, and September 30, 2021, early during the course of the COVID-19 pandemic; therefore, the generalizability of these findings to the current COVID-19 era may be limited. However, because the SARS-CoV-2 RNA-dependent RNA polymerase site that is targeted by remdesivir is conserved across SARS-CoV-2 variant eras^36^, the effectiveness of remdesivir is not expected to change over time. Because remdesivir was the only antiviral to receive EUA and subsequent approval for use in hospitalized patients in the United States during the study period, additional studies are needed to investigate any associations between the risk of Long COVID and the use of other antivirals that have since been authorized or approved. Timing of SARS-CoV-2 acquisition and Long COVID symptoms/diagnoses onset relative to hospitalization were not available, nor were provider/hospital practices related to remdesivir administration; this could have led to residual confounding. Additionally, the diagnostic modalities used to identify Long COVID symptoms were not captured in the study dataset; variation in diagnostic modalities likely contributed to heterogeneity in the clinical profile of the study population. Patient COVID-19 vaccination status was also unavailable, thus limiting the clinical significance of the findings of this study. Despite capturing the level of oxygen support and medications received during the first 2 days of hospitalization (including corticosteroids) and inverse probability weighting, residual confounding by severity is also possible. Dysautonomia, taste disturbance, and smell disturbance were rare, with ≤ 50 people experiencing the event in the overall population. The modeling results for these outcomes may be impacted by sparse data bias, which may result in extreme effect estimates and wide CIs^37^. The requirement of 270 days of follow-up may have introduced selection bias; however, prolonged follow-up was necessary to capture Long COVID outcomes. Because not all Long COVID outcomes prompt individuals to seek care (eg, when symptoms are mild or self-limiting) and not all symptoms/diagnoses may be coded during a medical encounter, the absolute incidence and impact of Long COVID outcomes are likely underestimated, which may explain the relatively low rate of reporting of certain outcomes that are prominent elsewhere in the literature, such as smell/taste disturbance^1,3,4^. The relatively small reduction in Long COVID was also affected by the inclusion of prespecified symptoms, several of which (eg, cough, joint pain, muscle pain) did not contribute to the risk, whereas others (eg, headache, diarrhea, cognitive symptoms, thrombosis) did consistently contribute; as a result, the effect of impactful outcomes is diluted by nonimpactful outcomes. On the other hand, Long COVID–related diagnoses could arise due to causes other than SARS-CoV-2 infection and may therefore be overestimated, particularly for preexisting comorbidities already prevalent by the time of index. There is no reason to expect that underreporting or overreporting of outcomes was differential between remdesivir-exposed and unexposed groups. Lastly, the ICD-10 code for Long COVID was introduced during the study period, affecting its reliability of use^38^. This was mitigated by a combined primary outcome, which included each of the individual Long COVID outcomes. Inclusion of a negative control group without a COVID-19 diagnosis in future studies could be helpful to contextualize the incidence of Long COVID outcomes.

Remdesivir exposure was associated with a modestly reduced risk of any Long COVID outcomes following hospitalization with COVID-19 in the overall study population and certain outcomes in patients with immunocompromising conditions. These data suggest a potential yet unidentified long-term benefit for hospitalized patients with COVID-19 treated with remdesivir. The results of this study highlight the vulnerability of patients with immunocompromising conditions to Long COVID, support further research into the outcomes following remdesivir treatment in the immunocompromised population for which benefit has been demonstrated^23^, and provide further support for the routine use of remdesivir in all patients hospitalized with COVID-19. Future analyses should account for vaccination, other COVID antivirals, antibody responses, and contemporary circulating variants, and explore the impact of remdesivir treatment on Long COVID across subpopulations, including those with different levels of oxygen support status and those hospitalized during different SARS-CoV-2 variant eras.

## Methods

### Data source

This retrospective cohort study used data from HealthVerity Real-time Insights and Evidence (Philadelphia, PA, USA), comprising hospital chargemaster data linked to closed medical and pharmacy claims (from commercial, Medicare, and Medicaid health plans) for inpatient and outpatient encounters across 50 states and includes > 25 million US patients. The study was approved and patient informed consent was waived under an applicable exemption for deidentified data by the WCG Institutional Review Board; the study followed the 2005 Guidelines for Good Epidemiologic Practice, Best Practices for Conducting and Reporting Pharmacoepidemiologic Safety Studies Using Electronic Healthcare Data Sets, and the 2015 International Society of Pharmacoepidemiology Good Pharmacoepidemiology Practices.

### Patients

The HealthVerity database was queried for patients ≥ 12 years of age admitted for hospitalization for ≥ 2 days with a diagnosis of COVID-19 (ICD-10 code U07.1) in any position (ie, nonprimary diagnoses were included) during the inpatient encounter between May 1, 2020 (when remdesivir received EUA from the US Food and Drug Administration), and September 30, 2021. Only the first hospitalization fulfilling all inclusion criteria was considered. Included patients had ≥ 365 days of continuous insurance enrollment prior to hospital admission through Day 2 of hospitalization (with a ≤ 30-day allowable gap) and survived the first 2 days of hospitalization. Exclusion criteria included diagnosis of Long COVID (ICD-10 code U09.9) prior to admission, pregnancy-related diagnoses during the hospitalization, and prior remdesivir use. Because manifestations of Long COVID could include exacerbation of ongoing disease, those experiencing symptoms consistent with Long COVID prior to admission were not excluded, and preexisting Long COVID–like symptoms were accounted for in propensity score weighting.

Additionally, 2 subgroups were identified: patients with a potentially immunocompromising condition (identified by ≥ 1 diagnosis code for HIV, hematologic or solid malignancy, organ transplantation, rheumatologic disease, or other immunosuppressive conditions during the ≤ 365 days before hospital admission)^29^ and patients with ≥ 1 moderately-to-severely immunocompromising therapy or condition (identified via an algorithm based on National Institutes of Health criteria; definitions for subgroups are summarized in Supplementary Table 4)^30^.

### Study design

The day of hospital admission was defined as Day 0; the index date was defined as Day 2. The remdesivir-exposed group included patients who received ≥ 1 dose of remdesivir on Day 0 or Day 1 based on chargemaster drug descriptions or procedure codes; the unexposed comparator group included patients with no evidence of remdesivir use during the first 2 days of hospitalization. Covariates for adjustment were measured in the baseline period from Day−365 to Day−1 (Supplementary Fig. 2). In an attempt to balance for preexisting disease states that could be mistaken for Long COVID in the primary assessment window postinfection and confound interpretation of Long COVID outcomes being measured, the baseline preinfection ICD-10 codes were assessed from Day –365 through Day –15. This baseline window concluded at Day –15 rather than Day –1 because some Long COVID outcomes overlap with acute infection symptoms and conditions.

For each Long COVID outcome, the outcome assessment period began 90 days after admission (consistent with the WHO definition of Long COVID^6^) until the earliest of the following: occurrence of the specific outcome of interest, inpatient death, disenrollment of insurance, data cutoff (April 30, 2022), or 270 days after admission. When a Long COVID outcome occurred, that patient was censored for that particular Long COVID outcome. However, follow-up continued for other potential Long COVID outcomes, and thus a single patient could contribute to ≥ 1 Long COVID outcome. For patients still hospitalized at Day 90, outcomes were assessed from the day after discharge through the end of follow-up.

Potential Long COVID outcomes were identified and defined based on physician coauthor input and a previously published systematic literature review and meta-analysis (including 52 studies) assessing Long COVID outcomes from 28 days to 1 year following COVID-19 hospital discharge^4^. The primary composite outcome of Long COVID occurred if patients had ≥ 1 ICD-10 diagnosis code (Supplementary Table 5) or ICD-10 code U09.9 (“post-COVID condition, unspecified”) in any position in claims or chargemaster data during the study assessment period. Secondary outcomes included each of the 16 Long COVID outcomes (neuropsychiatric features, dyspnea/breathlessness, fatigue, joint pain/arthralgia, cognitive dysfunction, chest pain, cerebrovascular disease, thromboembolic disease, cough, ischemic heart disease, diarrhea, headache, muscle pain/myalgia, dysautonomia, taste disturbance/dysgeusia/ageusia, or smell disturbance/anosmia). Outcomes occurring in the assessment period could be new onset (incident), ongoing from acute infection (persistent), or present in both the baseline and outcomes assessment time periods (prevalent). These outcome states are consistent with interpretations of WHO definitions of “new onset,” “persistent,” or “fluctuating” and emerging definitions from the RECOVER consortium focusing on prevalent symptoms after SARS-CoV-2 infection irrespective of their presence at baseline^6,39^.

### Statistical analysis

Descriptive analyses of baseline patient characteristics were performed across the full study population and compared between the 2 groups using absolute SMDs. To prevent bias that can arise from patients followed for different lengths of times, outcome event rates per 100 person-years were calculated as the number of patients experiencing the outcome divided by the total person-time contributed over the assessment period. The 95% CIs for the rates were calculated using the normal approximation.

In the primary analysis, a pseudo-population of patients who were remdesivir-exposed or unexposed, balanced for key characteristics, was created using weights derived from inverse probability weighting based on a propensity score for baseline characteristics, censoring, and competing risks (Supplementary Methods and Supplementary Table 6).

Log-binomial models were fit to estimate relative risks, 95% CIs (using the normal approximation using weighted standard errors), and *P* values for the composite outcome of any Long COVID outcome and for each of the 16 individual outcomes. Each outcome model only included patients who experienced the outcome of interest or had a complete 270 days of follow-up. Patients who were censored or had evidence of inpatient mortality contributed to weighting models but were not included in the outcome models; thus, the number of patients contributing to each model varied. The outcome models were weighted by the product of the 3 individual weights (treatment assignment, censoring, and competing risks)^40,41^. The 95% CIs and *P* values reported here were not adjusted for multiple comparisons; however, because of multiple comparisons, statistical significance was determined using the Holm-Bonferroni method (setting the significance threshold according to the number of tests performed) to reduce the likelihood of incorrectly rejecting the null hypothesis (type 1 error) rather than comparing the *P* values to an α of 0.05. The weighting and modeling process was conducted within each subgroup.

An as-treated sensitivity analysis (ie, considered the treatment received, irrespective of assignment) was performed by censoring patients who were unexposed by the index date and later received remdesivir at the point of crossover; crossover was incorporated into the weighting as a censoring event.

## Supplementary Information

Supplementary Information.

## Data Availability

Data are available for licensing from HealthVerity Real-time Insights and Evidence (Philadelphia, PA, USA). Please contact Mark Berry (Mark.Berry1@gilead.com) with data inquiries.

## References

[1] Davis, H. E., McCorkell, L., Vogel, J. M. & Topol, E. J. Long COVID: Major findings, mechanisms and recommendations. *Nat Rev Microbiol.* **21**, 133–146 (2023). 36639608 10.1038/s41579-022-00846-2 PMC9839201

[2] Bowe, B., Xie, Y. & Al-Aly, Z. Postacute sequelae of COVID-19 at 2 years. *Nat Med.* **29**, 2347–2357 (2023). 37605079 10.1038/s41591-023-02521-2 PMC10504070

[3] O’Mahoney, L. L. et al. The prevalence and long-term health effects of Long Covid among hospitalised and non-hospitalised populations: A systematic review and meta-analysis. *EClinicalMedicine.* **55**, 101762 (2023). 36474804 10.1016/j.eclinm.2022.101762 PMC9714474

[4] Kelly, J. D. et al. SARS-CoV-2 post-acute sequelae in previously hospitalised patients: Systematic literature review and meta-analysis. *Eur Respir Rev.* **32**, 220254 (2023). 37437914 10.1183/16000617.0254-2022 PMC10336551

[5] National Academies of Sciences, Engineering, and Medicine. *A Long COVID Definition: A Chronic, Systemic Disease State with Profound Consequences* (The National Academies Press, Washington, DC, 2024). 10.17226/27768. 39110819

[6] Soriano, J. B. et al. A clinical case definition of post-COVID-19 condition by a Delphi consensus. *Lancet Infect Dis.* **22**, e102–e107 (2022). 34951953 10.1016/S1473-3099(21)00703-9 PMC8691845

[7] US Department of Health and Human Services. What is long COVID? 2022 [updated 2022/12/16].

[8] Ioannou, G. N. et al. Rates and factors associated with documentation of diagnostic codes for long COVID in the National Veterans Affairs Health Care System. *JAMA Netw Open.* **5**, e2224359 (2022). 35904783 10.1001/jamanetworkopen.2022.24359 PMC9338411

[9] Global Burden of Disease Long COVID Collaborators et al. Estimated global proportions of individuals with persistent fatigue, cognitive, and respiratory symptom clusters following symptomatic COVID-19 in 2020 and 2021. *JAMA.* **328,** 1604–1615 (2022). 10.1001/jama.2022.18931 PMC9552043 36215063

[10] DeWolf, S. et al. SARS-CoV-2 in immunocompromised individuals. *Immunity* **55**, 1779–1798 (2022). 36182669 10.1016/j.immuni.2022.09.006 PMC9468314

[11] Wang, Q., Berger, N. A. & Xu, R. Analyses of risk, racial disparity, and outcomes among US patients with cancer and COVID-19 infection. *JAMA Oncol.* **7**, 220–227 (2021). 33300956 10.1001/jamaoncol.2020.6178 PMC7729584

[12] Goldman, J. D., Robinson, P. C., Uldrick, T. S. & Ljungman, P. COVID-19 in immunocompromised populations: Implications for prognosis and repurposing of immunotherapies. *J Immunother Cancer.* **9**, e002630 (2021). 34117116 10.1136/jitc-2021-002630 PMC8206176

[13] Su, Y. et al. Multiple early factors anticipate post-acute COVID-19 sequelae. *Cell* **185**, 881-895.e820 (2022). 35216672 10.1016/j.cell.2022.01.014 PMC8786632

[14] Swank, Z. et al. Persistent circulating severe acute respiratory syndrome coronavirus 2 spike is associated with post-acute coronavirus disease 2019 sequelae. *Clin Infect Dis.* **76**, e487–e490 (2023). 36052466 10.1093/cid/ciac722 PMC10169416

[15] Xie, Y., Choi, T. & Al-Aly, Z. Association of treatment with nirmatrelvir and the risk of post-COVID-19 condition. *JAMA Intern Med.* **183**, 554–564 (2023). 36951829 10.1001/jamainternmed.2023.0743 PMC10037200

[16] Fung, K. W., Baye, F., Baik, S. H. & McDonald, C. J. Nirmatrelvir and molnupiravir and post-COVID-19 condition in older patients. *JAMA Intern Med.* **183**, 1404–1406 (2023). 37870856 10.1001/jamainternmed.2023.5099 PMC10594174

[17] Boglione, L. et al. Risk factors and incidence of long-COVID syndrome in hospitalized patients: Does remdesivir have a protective effect?. *QJM* **114**, 865–871 (2022). 34850210 10.1093/qjmed/hcab297 PMC8690187

[18] Nevalainen, O. P. O. et al. Effect of remdesivir post hospitalization for COVID-19 infection from the randomized SOLIDARITY Finland trial. *Nat Commun.* **13**, 6152 (2022). 36257950 10.1038/s41467-022-33825-5 PMC9579198

[19] Beigel, J. H. et al. Remdesivir for the treatment of Covid-19: Final report. *N Engl J Med.* **383**, 1813–1826 (2020). 32445440 10.1056/NEJMoa2007764 PMC7262788

[20] Spinner, C. D. et al. Effect of remdesivir vs standard care on clinical status at 11 days in patients with moderate COVID-19: A randomized clinical trial. *JAMA* **324**, 1048–1057 (2020). 32821939 10.1001/jama.2020.16349 PMC7442954

[21] Gottlieb, R. L. et al. Early remdesivir to prevent progression to severe Covid-19 in outpatients. *N Engl J Med.* **386**, 305–315 (2022). 34937145 10.1056/NEJMoa2116846 PMC8757570

[22] Amstutz, A. et al. Effects of remdesivir in patients hospitalised with COVID-19: A systematic review and individual patient data meta-analysis of randomised controlled trials. *Lancet Respir Med.* **11**, 453–464 (2023). 36828006 10.1016/S2213-2600(22)00528-8 PMC10156140

[23] Mozaffari, E. et al. Remdesivir reduced mortality in immunocompromised patients hospitalized for COVID-19 across variant waves: Findings from routine clinical practice. *Clin Infect Dis.* **77**, 1626–1634 (2023). 37556727 10.1093/cid/ciad460 PMC10724457

[24] Eastman, R. T. et al. Remdesivir: A review of its discovery and development leading to emergency use authorization for treatment of COVID-19. *ACS Cent Sci.* **6**, 672–683 (2020). 32483554 10.1021/acscentsci.0c00489 PMC7202249

[25] COVID-19 Treatment Guidelines Panel. Coronavirus disease 2019 (COVID-19) treatment guidelines. https://www.ncbi.nlm.nih.gov/books/NBK570371/pdf/Bookshelf_NBK570371.pdf

[26] Bhimraj, A. et al. Infectious Diseases Society of America guidelines on the treatment and management of patients with COVID-19. *Clin Infect Dis.* **78**, ciac724 (2022). 10.1093/cid/ciac724 PMC9494372 36063397

[27] Rajme-López, S. et al. Early outpatient treatment with remdesivir in patients at high risk for severe COVID-19: A prospective cohort study. *Open Forum Infect Dis.* **9**, ofac502 (2022). 36285176 10.1093/ofid/ofac502 PMC9585545

[28] Lafont, E. et al. Targeted SARS-CoV-2 treatment is associated with decreased mortality in immunocompromised patients with COVID-19. *J Antimicrob Chemother.* **77**, 2688–2692 (2022). 35876174 10.1093/jac/dkac253 PMC9384481

[29] Patel, M. et al. Analysis of MarketScan data for immunosuppressive conditions and hospitalizations for acute respiratory illness, United States. *Emerg Infect Dis.* **26**, 1720–1730 (2020). 32348234 10.3201/eid2608.191493 PMC7392442

[30] US Centers for Disease Control and Prevention. People who are immunocompromised 2023 [updated 2023/05/11]. https://archive.cdc.gov/www_cdc_gov/coronavirus/2019-ncov/need-extra-precautions/people-who-are-immunocompromised.html.

[31] WHO Solidarity Trial Consortium. Remdesivir and three other drugs for hospitalised patients with COVID-19: Final results of the WHO Solidarity randomised trial and updated meta-analyses. *Lancet*. **399**, 1941–1953 (2022). 35512728 10.1016/S0140-6736(22)00519-0 PMC9060606

[32] Lee, T. C. et al. Remdesivir for the treatment of COVID-19: A systematic review and meta-analysis. *Clin Microbiol Infect.* **28**, 1203–1210 (2022). 35598856 10.1016/j.cmi.2022.04.018 PMC9117160

[33] Chokkalingam, A. P. et al. Association of remdesivir treatment with mortality among hospitalized adults with COVID-19 in the United States. *JAMA Netw Open.* **5**, e2244505 (2022). 36454570 10.1001/jamanetworkopen.2022.44505 PMC9716380

[34] Mozaffari, E. et al. Remdesivir treatment in hospitalized patients with coronavirus disease 2019 (COVID-19): A comparative analysis of in-hospital all-cause mortality in a large multicenter observational cohort. *Clin Infect Dis.* **75**, e450–e458 (2022). 34596223 10.1093/cid/ciab875 PMC9402660

[35] Mozaffari, E. et al. Remdesivir is associated with reduced mortality in COVID-19 patients requiring supplemental oxygen including invasive mechanical ventilation across SARS-CoV-2 variants. *Open Forum Infect Dis.* **10**, ofad482 (2023). 37869410 10.1093/ofid/ofad482 PMC10588622

[36] Martin, R. et al. Genetic conservation of SARS-CoV-2 RNA replication complex in globally circulating isolates and recently emerged variants from humans and minks suggests minimal pre-existing resistance to remdesivir. *Antiviral Res.* **188**, 105033 (2021). 33549572 10.1016/j.antiviral.2021.105033 PMC7862048

[37] Greenland, S., Mansournia, M. A. & Altman, D. G. Sparse data bias: A problem hiding in plain sight. *BMJ* **352**, i1981 (2016). 27121591 10.1136/bmj.i1981

[38] Pfaff, E. R. et al. Coding long COVID: Characterizing a new disease through an ICD-10 lens. *BMC Med.* **21**, 58 (2023). 36793086 10.1186/s12916-023-02737-6 PMC9931566

[39] Thaweethai, T. et al. Development of a definition of postacute sequelae of SARS-CoV-2 infection. *JAMA*. **329**, 1934–1946 (2023). 37278994 10.1001/jama.2023.8823 PMC10214179

[40] Finn, A., Jindal, A., Andrea, S. B., Selvaraj, V. & Dapaah-Afriyie, K. Association of treatment with remdesivir and 30-day hospital readmissions in patients hospitalized with COVID-19. *Am J Med Sci.* **363**, 403–410 (2022). 35151637 10.1016/j.amjms.2022.01.021 PMC8830144

[41] Xie, D. et al. Statistical methods for modeling time-updated exposures in cohort studies of chronic kidney disease. *Clin J Am Soc Nephrol.* **12**, 1892–1899 (2017). 28818846 10.2215/CJN.00650117 PMC5672960

