# Supplementary material for: Risk of Long COVID in hospitalized individuals treated with remdesivir for acute COVID-19
Source: Sci Rep. 2025 Jul 28;15:27441. doi: 10.1038/s41598-025-06052-3 (PMC12304269; doi:10.1038/s41598-025-06052-3)
Supplement: Supplementary file 1 — Supplementary Information. [file 41598_2025_6052_MOESM1_ESM.docx]

# SUPPLEMENTARY APPENDIX

Supplement to: Mark Berry, Amanda M. Kong, Roger Paredes, Julie Paone, Rohan Shah, Rebecca Taylor, Essy Mozaffari, Rikisha Gupta, Robert L. Gottlieb, Lourdes Mateu, Mazin Abdelghany, Jason D. Goldman, Anand P. Chokkalingam. Risk of Long COVID in Hospitalized Individuals Treated With Remdesivir for Acute COVID-19: A Retrospective Cohort Study

**Supplementary Methods**

In the primary analysis, a pseudo-population of remdesivir-exposed and unexposed patients balanced for key characteristics was created using inverse probability of treatment weights, inverse probability of censoring (ie, disenrollment from insurance) weights, and inverse probability of competing risks (ie, inpatient mortality) weights. Weights were derived using a logistic regression accounting for calendar time of hospitalization, demographics, comorbidities, concomitant medications, COVID-19 severity, and presence/absence of each of the investigated Long COVID outcomes at baseline (**Supplementary** **Table 6**). Comorbidities were defined by aggregates of *International Classification of Diseases, Tenth Revision, Clinical Modification* (ICD-10-CM)/ Procedure Coding System (PCS)^1^ and did not include diagnosis codes for Long COVID. For competing risks, patients without evidence of inpatient mortality that resemble patients with evidence of inpatient mortality were assigned greater weight to approximate how the population would have looked if all patients had complete follow-up.

Propensity scores were calculated with data from the full study population using a logistic regression model with exposure to remdesivir (between Day 0 and Day 2) as the dependent variable and observed baseline characteristics as independent variables. Similar methods were used to generate the weights to account for censoring and competing risks, including an additional binary indicator for remdesivir exposure. Each weight was computed separately assuming that the baseline covariates (and treatment indicator), censoring, competing risks, and treatment indicator were independent. Because patient follow-up continued after a patient was censored for any single Long COVID outcome, and a patient could be censored or die in the analysis of one outcome but not another, the weighting process was conducted separately for each outcome. Each set of weights was stabilized following the process described by Hernan and Robins and truncated at 10 to minimize the presence of extreme weights (<0.3% of the weights were truncated for the overall population).^2^ After weighting, balance in baseline characteristics was assessed using absolute standardized mean differences.

Aetion and specific Gilead authors had access to the full dataset used to generate the analytic cohort in this analysis via the Aetion^®^ Substantiate platform. Minimal data cleaning was performed, including removal of events with no associated dates and individuals with missing age.

**Supplementary Figures**

# Supplementary Figure 1

**Supplementary Figure 1. Association of remdesivir with Long COVID-associated outcomes after hospitalization with COVID-19 in the as-treated population.**
The weighted risk of Long COVID outcomes associated with remdesivir administration compared with no remdesivir administration is shown in descending order of total events for the as-treated population. This sensitivity analysis was performed by censoring unexposed patients who later crossed over (received remdesivir) at the point of crossover. Abbreviations: RR, risk ratio. *Statistically significant outcomes after adjusting statistical inference for multiple comparisons testing using the Holm-Bonferroni method (total α = 0.05; m = 17, where m is the number of hypotheses). ^†^Error bars for smell disturbance/anosmia extend beyond the shown scale.

# Supplementary Figure 2

**
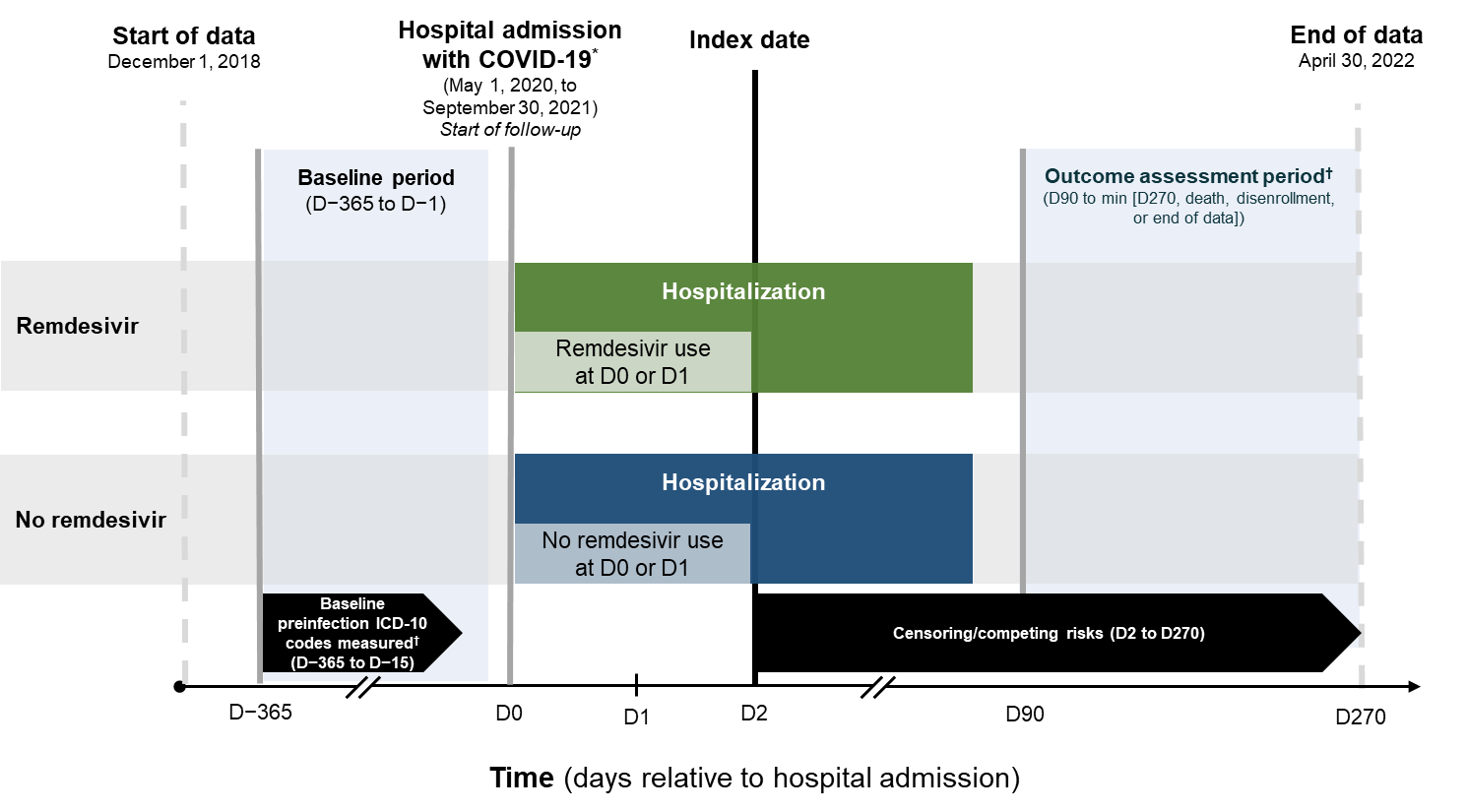
**

**Supplementary Figure 2.** **Assessment periods for Long COVID-associated outcomes of individuals hospitalized with a diagnosis of COVID-19.**
Baseline preinfection ICD-10 codes were measured from D −365 to D −15 to capture overlap of some Long COVID outcomes with acute infection symptoms and conditions. Abbreviations: D, day relative to hospital admission; ICD-10, *International Classification of Diseases, Tenth Revision*; min, minimum; WHO, World Health Organization.

^*^Admitted with COVID-19 (ICD-10 code U07.1) in any position during an inpatient encounter in a medical claim or chargemaster record. ^†^The study outcome assessment period started 90 days after hospital admission (consistent with the WHO definition of Long COVID).^3^ Because some Long COVID outcomes overlap with acute infection symptoms and conditions, baseline preinfection ICD-10 codes were measured from Day −365 to Day −15. Outcomes occurring in the assessment period could be new onset (incident), ongoing from acute infection (persistent), or present in both the baseline and outcomes assessment time periods (prevalent).

# Supplementary Table 1. Patients With a Moderately-to-Severely Immunocompromising Therapy or Condition^*^ During the Baseline Period

| **Characteristic** | **Remdesivir-exposed**  **(n = 17,164)** | **Unexposed**  **(n = 34,842)** | **Total**  **(N = 52,006)** |
| --- | --- | --- | --- |
| **Moderately-to-severely immunocompromised**, n (%) | 1686 (9.8) | 3987 (11.4) | 5673 (10.9) |
| Treatment with ≥2 high-dose steroids^†^  (1-180 days before hospitalization) | 1245 (73.8) | 2775 (69.6) | 4020 (70.9) |
| Active treatment for solid tumor and hematologic malignancies (1-180 days before hospitalization) | 342 (20.3) | 935 (23.5) | 1277 (22.5) |
| Advanced HIV infection (1-180 days before hospitalization) or untreated HIV infection  (1-365 days before hospitalization) | 69 (4.1) | 218 (5.5) | 287 (5.1) |
| CAR-T or HCT therapy (1-730 days before hospitalization)^‡^ | 14 (0.8) | 31 (0.8) | 45 (0.8) |
| Moderate or severe primary immunodeficiency (any time prior to 1 day before hospitalization) | 97 (5.8) | 234 (5.9) | 331 (5.8) |
| Solid-organ transplantation (1-180 days before hospitalization) | 140 (8.3) | 399 (10.0) | 539 (9.5) |

CAR-T, chimeric antigen receptor T; HCT, hematopoietic cell transplantation; IV, intravenous; NIH, National Institutes of Health.

^*^Immunocompromised by modified NIH definition (**Supplementary Table 4**).^4^

^†^IV corticosteroid or oral corticosteroids (prednisone, methylprednisolone, hydrocortisone, or dexamethasone) with a dose of ≥20 mg (or equivalent).

^‡^Or CAR-T or HCT (any time prior to 1 day before hospitalization) plus immunosuppressive therapy (1-180 days before hospitalization).

# Supplementary Table 2. Reasons for the End-of-data Collection Across the Overall Population

|  | **Remdesivir-exposed**  **(n = 17,164)** | | | | **Unexposed**  **(n = 34,842)** | | | |
| --- | --- | --- | --- | --- | --- | --- | --- | --- |
| **Outcome** | **Inpatient death** | **Censored** | **Completed 270 days of follow-up** | **Outcome** | **Inpatient death** | **Censored** | **Completed 270 days of follow-up** | **Outcome** |
| Any Long COVID outcome | 302 | 2437 | 5782 | 8643 | 945 | 5803 | 7892 | 20,202 |
| Neuropsychiatric features | 317 | 2739 | 9692 | 4416 | 1062 | 6760 | 14,559 | 12,461 |
| Dyspnea/breathlessness | 331 | 2876 | 11,248 | 2709 | 1092 | 7336 | 20,622 | 5792 |
| Fatigue | 335 | 2895 | 11,640 | 2294 | 1108 | 7288 | 20,507 | 5939 |
| Joint pain/arthralgia | 354 | 2948 | 11,362 | 2500 | 1198 | 7545 | 20,795 | 5304 |
| Cognitive dysfunction | 336 | 2922 | 12,502 | 1404 | 1061 | 7137 | 20,818 | 5826 |
| Chest pain | 337 | 2964 | 12,046 | 1817 | 1144 | 7537 | 21,256 | 4905 |
| Cerebrovascular disease | 341 | 2977 | 12,807 | 1039 | 1144 | 7432 | 22,358 | 3908 |
| Thromboembolic disease | 339 | 2994 | 12,943 | 888 | 1163 | 7669 | 23,595 | 2415 |
| Cough | 349 | 3015 | 12,791 | 1009 | 1201 | 7698 | 23,399 | 2544 |
| Ischemic heart disease | 329 | 3010 | 13,187 | 638 | 1150 | 7663 | 23,914 | 2115 |
| Diarrhea | 353 | 3035 | 13,343 | 433 | 1209 | 7796 | 24,249 | 1588 |
| Headache | 356 | 3036 | 13,416 | 356 | 1234 | 7858 | 24,710 | 1040 |
| Muscle pain/myalgia | 358 | 3055 | 13,343 | 408 | 1236 | 7870 | 24,910 | 826 |
| Dysautonomia | 358 | 3069 | 13,730 | 7 | 1238 | 7923 | 25,638 | 43 |
| Taste disturbance/dysgeusia/ageusia | 358 | 3069 | 13,725 | 12 | 1242 | 7926 | 25,646 | 28 |
| Smell disturbance/anosmia | 358 | 3068 | 13,715 | 23 | 1242 | 7927 | 25,650 | 23 |

# Supplementary Table 3. Absolute Standardized Mean Differences (SMD) for All Outcome Models with Remdesivir Treatment

| **Outcome Model** | | | | | | | | |
| --- | --- | --- | --- | --- | --- | --- | --- | --- |
| **Any Long COVID Outcome** | | **Neuropsychiatric features** | | **Muscle pain/myalgia** | | **Taste disturbance/dysgeusia/ageusia** | |  |
| **Variable** | **SMD** | **Variable** | **SMD** | **Variable** | **SMD** | **Variable** | **SMD** |  |
| Day 1 or Day 2: Corticosteroid use | 0.24 | Day 1 or Day 2: Corticosteroid use | 0.23 | Day 1 or Day 2: Corticosteroid use | 0.23 | Day 1 or Day 2: Corticosteroid use | 0.23 |  |
| Index date: May 2020 | 0.20 | Index date: May 2020 | 0.19 | Index date: May 2020 | 0.19 | Index date: May 2020 | 0.19 |  |
| Index date: June 2020 | 0.10 | Index date: June 2020 | 0.10 | Index date: June 2020 | 0.10 | Index date: June 2020 | 0.10 |  |
| Previous COVID-19 infection | 0.10 | Previous COVID-19 infection | 0.10 | Previous COVID-19 infection | 0.10 | Previous COVID-19 infection | 0.10 |  |
| Mood/neuropsychiatric features | 0.08 | Mood/neuropsychiatric features | 0.09 | Mood/neuropsychiatric features | 0.09 | Mood/neuropsychiatric features | 0.09 |  |
| Index date: July 2020 | 0.08 | Index date: July 2020 | 0.08 | Index date: July 2020 | 0.09 | Index date: July 2020 | 0.09 |  |
| Cognitive dysfunction | 0.07 | Cognitive dysfunction | 0.07 | Neuropsychiatric features | 0.08 | Neuropsychiatric features | 0.08 |  |
| Neuropsychiatric features | 0.06 | Neuropsychiatric features | 0.07 | Index date: December 2020 | 0.07 | Index date: December 2020 | 0.07 |  |
| Index date: December 2020 | 0.06 | Index date: December 2020 | 0.06 | Cognitive dysfunction | 0.06 | Cognitive dysfunction | 0.06 |  |
| Infection (non–COVID-19) | 0.06 | Infection (non–COVID-19) | 0.06 | Day 1 or Day 2: Low-flow oxygen | 0.06 | Day 1 or Day 2: Low-flow oxygen | 0.06 |  |
| Heart failure | 0.05 | Day 1 or Day 2: Low-flow oxygen | 0.05 | Infection (non–COVID-19) | 0.05 | Infection (non–COVID-19) | 0.06 |  |
| Day 1 or Day 2: Low-flow oxygen | 0.05 | Heart failure | 0.05 | Index date: January 2021 | 0.05 | Day 1 or Day 2: High-flow oxygen | 0.05 |  |
| Index date: January 2021 | 0.05 | Day 1 or Day 2: High-flow oxygen | 0.05 | Day 1 or Day 2: High-flow oxygen | 0.05 | Index date: January 2021 | 0.05 |  |
| Day 1 or Day 2: High-flow oxygen | 0.05 | Index date: January 2021 | 0.05 | Heart failure | 0.05 | Chest pain | 0.05 |  |
| Blood condition | 0.05 | Chest pain | 0.05 | Chest pain | 0.05 | Heart failure | 0.05 |  |
| Chest pain | 0.04 | Cerebrovascular disease | 0.05 | Smoking | 0.05 | Smoking | 0.05 |  |
| Index date: April 2021 | 0.04 | Blood condition | 0.05 | Genitourinary condition | 0.04 | Genitourinary condition | 0.04 |  |
| Cerebrovascular disease | 0.04 | Index date: April 2021 | 0.04 | Blood condition | 0.04 | Renal disease (acute) | 0.04 |  |
| Abnormal blood pressure (hypo) | 0.04 | Day 1 or Day 2: Anticoagulant use | 0.04 | Index date: April 2021 | 0.04 | Blood condition | 0.04 |  |
| Renal disease (acute) | 0.04 | Genitourinary condition | 0.04 | Renal disease (acute) | 0.04 | Index date: April 2021 | 0.04 |  |
| Genitourinary condition | 0.04 | Renal disease (acute) | 0.04 | Day 1 or Day 2: Convalescent plasma use | 0.04 | Age | 0.04 |  |
| Day 1 or Day 2: Anticoagulant use | 0.04 | Smoking | 0.04 | Other forms of heart disease | 0.04 | Day 1 or Day 2: Convalescent plasma use | 0.04 |  |
| Conductive disorders, dysrhythmias | 0.04 | Abnormal blood pressure (hypo) | 0.04 | Cerebrovascular disease | 0.04 | Cerebrovascular disease | 0.04 |  |
| Other forms of heart disease | 0.04 | Other forms of heart disease | 0.04 | Age | 0.04 | Other forms of heart disease | 0.04 |  |
| Thromboembolic disease | 0.04 | Region: South | 0.04 | Ischemic heart disease | 0.04 | Ischemic heart disease | 0.04 |  |
| Diseases of arteries, arterioles, and capillaries | 0.04 | Day 1 or Day 2: Convalescent plasma use | 0.04 | Day 1 or Day 2: Anticoagulant use | 0.04 | Day 1 or Day 2: Anticoagulant use | 0.04 |  |
| Day 1 or Day 2: Convalescent plasma use | 0.03 | Diseases of arteries, arterioles, and capillaries | 0.04 | Thromboembolic disease | 0.04 | Headache | 0.04 |  |
| Index date: November 2020 | 0.03 | Thromboembolic disease | 0.04 | Headache | 0.03 | Day 1 or Day 2: Immunomodulator use | 0.03 |  |
| Behavioral condition | 0.03 | Conductive disorders, dysrhythmias | 0.03 | Behavioral condition | 0.03 | Thromboembolic disease | 0.03 |  |
| Day 1 or Day 2: Immunomodulator use | 0.03 | Behavioral condition | 0.03 | Day 1 or Day 2: Immunomodulator use | 0.03 | Behavioral condition | 0.03 |  |
| Ischemic heart disease | 0.03 | Day 1 or Day 2: Immunomodulator use | 0.03 | Region: South | 0.03 | Abnormal blood pressure (hypo) | 0.03 |  |
| Index date: August 2020 | 0.03 | Age | 0.03 | Diseases of arteries, arterioles, and capillaries | 0.03 | Region: South | 0.03 |  |
| Cough | 0.03 | Ischemic heart disease | 0.03 | Conductive disorders, dysrhythmias | 0.03 | Day 1 or Day 2: ICU admission | 0.03 |  |
| Other ischemic heart disease | 0.03 | Index date: November 2020 | 0.03 | Abnormal blood pressure (hypo) | 0.03 | Diseases of arteries, arterioles, and capillaries | 0.03 |  |
| Day 1 or Day 2: ICU admission | 0.03 | Circulatory diseases | 0.03 | Region: Northeast | 0.03 | Region: Northeast | 0.03 |  |
| Circulatory disease | 0.03 | Dyspnea/breathlessness | 0.03 | Digestive condition | 0.03 | Conductive disorders, dysrhythmias | 0.03 |  |
| Dyspnea/breathlessness | 0.03 | Cough | 0.03 | Day 1 or Day 2: ICU admission | 0.03 | Digestive condition | 0.03 |  |
| Fatigue | 0.03 | Day 1 or Day 2: ICU admission | 0.03 | Cough | 0.03 | Dyspnea/breathlessness | 0.03 |  |
| Digestive condition | 0.03 | Region: Northeast | 0.03 | Dyspnea/breathlessness | 0.03 | Cough | 0.03 |  |
| Smoking | 0.03 | Digestive condition | 0.03 | Circulatory diseases | 0.03 | Index date: November 2020 | 0.02 |  |
| Region: Northeast | 0.03 | Other ischemic heart disease | 0.03 | Index date: November 2020 | 0.02 | Circulatory diseases | 0.02 |  |
| Region South | 0.03 | Index date: August 2020 | 0.03 | Factors influencing health status | 0.02 | Other signs/symptoms | 0.02 |  |
| Headache | 0.03 | Fatigue | 0.03 | Other signs/symptoms | 0.02 | Fatigue | 0.02 |  |
| Age | 0.03 | Headache | 0.03 | Other ischemic heart disease | 0.02 | Factors influencing health status | 0.02 |  |
| Factors influencing health status | 0.02 | Other and unspecified disorders of the circulatory system | 0.03 | Fatigue | 0.02 | Obesity | 0.02 |  |
| Other and unspecified disorders of the circulatory system | 0.02 | Other signs/symptoms | 0.02 | Obesity | 0.02 | Other ischemic heart disease | 0.02 |  |
| Other signs/symptoms | 0.02 | Obesity | 0.02 | Endocrine condition | 0.02 | Neurologic condition | 0.02 |  |
| Obesity | 0.02 | Factors influencing health status | 0.02 | Neurologic condition | 0.02 | Endocrine condition | 0.02 |  |
| Neoplasms | 0.02 | Neurologic condition | 0.02 | Index date: August 2020 | 0.02 | Diseases of veins, lymphatic vessels, and lymph nodes, not elsewhere classified | 0.02 |  |
| Index date: September 2021 | 0.02 | Neoplasms | 0.02 | Smell disturbance/anosmia | 0.02 | Index date: August 2020 | 0.02 |  |
| Diarrhea | 0.02 | Diarrhea | 0.02 | Diarrhea | 0.02 | Smell disturbance/anosmia | 0.02 |  |
| Index date: August 2021 | 0.02 | Index date: September 2021 | 0.02 | Other and unspecified disorders of the circulatory system | 0.02 | Index date: September 2021 | 0.02 |  |
| Endocrine condition | 0.02 | Diseases of veins, lymphatic vessels, and lymph nodes, not elsewhere classified | 0.02 | Diseases of veins, lymphatic vessels, and lymph nodes, not elsewhere classified | 0.02 | Other and unspecified disorders of the circulatory system | 0.02 |  |
| Diabetes | 0.02 | Index date: February 2021 | 0.02 | Index date: September 2021 | 0.02 | Diarrhea | 0.02 |  |
| Index date: February 2021 | 0.02 | Smell disturbance/anosmia | 0.02 | Neoplasms | 0.02 | Day 1 or Day 2: Protease inhibitor use | 0.02 |  |
| Day 1 or Day 2: Protease inhibitor use | 0.02 | Endocrine condition | 0.02 | Day 1 or Day 2: Protease inhibitor use | 0.02 | Index date: August 2021 | 0.02 |  |
| Diseases of veins, lymphatic vessels, and lymph nodes, not elsewhere classified | 0.02 | Diabetes | 0.02 | Index date: August 2021 | 0.02 | Neoplasms | 0.02 |  |
| Region: West | 0.01 | Day 1 or Day 2: Protease inhibitor use | 0.02 | Region: West | 0.01 | Region: West | 0.01 |  |
| Neurologic condition | 0.01 | Index date: August 2021 | 0.01 | Index date: October 2020 | 0.01 | Joint pain/arthralgia | 0.01 |  |
| Smell disturbance/anosmia | 0.01 | Musculoskeletal/connective tissue conditions | 0.01 | Diabetes | 0.01 | Index date: October 2020 | 0.01 |  |
| Renal disease: Chronic | 0.01 | Dysautonomia | 0.01 | Joint pain/arthralgia | 0.01 | Diabetes | 0.01 |  |
| Index date: May 2021 | 0.01 | Joint pain/arthralgia | 0.01 | Abnormal blood pressure (hyper) | 0.01 | Index date: February 2021 | 0.01 |  |
| Joint pain/arthralgia | 0.01 | Index date: October 2020 | 0.01 | Malformations | 0.01 | Abnormal blood pressure (hyper) | 0.01 |  |
| Abnormal blood pressure (hyper) | 0.01 | Index date: May 2021 | 0.01 | Index date: February 2021 | 0.01 | Dysautonomia | 0.01 |  |
| Musculoskeletal (connective tissue) | 0.01 | Region: West | 0.01 | Dysautonomia | 0.01 | Region: Midwest | 0.01 |  |
| Region: Midwest | 0.01 | Abnormal blood pressure (hyper) | 0.01 | Index date: May 2021 | 0.01 | Malformations | 0.01 |  |
| Index date: October 2020 | 0.01 | Renal disease: chronic | 0.01 | Muscle pain/myalgia | 0.01 | Evidence of organ transplant | 0.01 |  |
| Dysautonomia | 0.01 | Region: Midwest | 0.01 | Respiratory condition | 0.01 | Respiratory condition | 0.01 |  |
| Diseases of pulmonary circulation | 0.01 | Respiratory condition | 0.01 | Evidence of organ transplant | 0.01 | Musculoskeletal/connective tissue conditions | 0.01 |  |
| Respiratory condition | 0.01 | Index date: March 2021 | 0.01 | Region: Midwest | 0.01 | Renal chronic | 0.01 |  |
| Index date: July 2021 | 0.01 | Taste disturbance/dysgeusia/ageusia | 0.01 | Renal chronic | 0.01 | Taste disturbance/dysgeusia/ageusia | 0.01 |  |
| Index date: March 2021 | 0.01 | Diseases of pulmonary circulation | 0.01 | Musculoskeletal/connective tissue conditions | 0.01 | Muscle pain/myalgia | 0.01 |  |
| Taste disturbance/dysgeusia/ageusia | 0.00 | Metabolic condition | 0.01 | Taste disturbance/dysgeusia/ageusia | 0.01 | Index date: June 2021 | 0.01 |  |
| Metabolic condition | 0.00 | Index date: July 2021 | 0.00 | Diseases of pulmonary circulation | 0.01 | Diseases of pulmonary circulation | 0.01 |  |
| Malformations | 0.00 | Malformations | 0.00 | Index date: June 2021 | 0.01 | Day 1 or Day 2: ECMO/invasive mechanical ventilation | 0.00 |  |
| Index date: June 2021 | 0.00 | Rheumatic fever | 0.00 | Day 1 or Day 2: ECMO/invasive mechanical ventilation | 0.00 | Index date: May 2021 | 0.00 |  |
| Index date: September 2020 | 0.00 | Index date: September 2020 | 0.00 | Index date: September 2020 | 0.00 | Index date: September 2020 | 0.00 |  |
| Rheumatic fever | 0.00 | Day 1 or Day 2: ECMO/invasive mechanical ventilation | 0.00 | Sex: Female | 0.00 | Index date: March 2021 | 0.00 |  |
| Muscle pain/myalgia | 0.00 | Index date: October 2021 | 0.00 | Rheumatic fever | 0.00 | Sex: Female | 0.00 |  |
| Evidence of organ transplant | 0.00 | Index date: June 2021 | 0.00 | Index date: March 2021 | 0.00 | Index date: July 2021 | 0.00 |  |
| Sex: Female | 0.00 | Evidence of organ transplant | 0.00 | Index date: July 2021 | 0.00 | Rheumatic fever | 0.00 |  |
| Index date: October 2021 | 0.00 | Muscle pain/myalgia | 0.00 | Metabolic condition | 0.00 | Index date: October 2021 | 0.00 |  |
| Day 1 or Day 2: ECMO/invasive mechanical ventilation | 0.00 | Sex: Female | 0.00 | Index date: October 2021 | 0.00 | Metabolic condition | 0.00 |  |
|  |  |  |  |  |  |  |  |  |
| **Smell disturbance/anosmia** | | **Joint pain/arthralgia** | | **Ischemic heart disease** | | **Headache** | |  |
| **Variable** | **SMD** | **Variable** | **SMD** | **Variable** | **SMD** | **Variable** | **SMD** |  |
| Day 1 or Day 2: Corticosteroid use | 0.23 | Day 1 or Day 2: Corticosteroid use | 0.23 | Day 1 or Day 2: Corticosteroid use | 0.23 | Day 1 or Day 2: Corticosteroid use | 0.23 |  |
| Index date: May 2020 | 0.19 | Index date: May 2020 | 0.19 | Index date: May 2020 | 0.19 | Index date: May 2020 | 0.19 |  |
| Index date: June 2020 | 0.10 | Index date: June 2020 | 0.10 | Index date: June 2020 | 0.10 | Index date: June 2020 | 0.10 |  |
| Previous COVID-19 infection | 0.10 | Previous COVID-19 infection | 0.09 | Previous COVID-19 infection | 0.10 | Mood/neuropsychiatric features | 0.09 |  |
| Mood/neuropsychiatric features | 0.09 | Mood/neuropsychiatric features | 0.09 | Mood/neuropsychiatric features | 0.09 | Previous COVID-19 infection | 0.09 |  |
| Index date: July 2020 | 0.09 | Index date: July 2020 | 0.08 | Index date: July 2020 | 0.09 | Index date: July 2020 | 0.09 |  |
| Neuropsychiatric features | 0.08 | Neuropsychiatric features | 0.08 | Neuropsychiatric features | 0.07 | Neuropsychiatric features | 0.07 |  |
| Index date: December 2020 | 0.07 | Index date: December 2020 | 0.07 | Index date: December 2020 | 0.07 | Index date: December 2020 | 0.07 |  |
| Cognitive dysfunction | 0.06 | Cognitive dysfunction | 0.06 | Cognitive dysfunction | 0.06 | Cognitive dysfunction | 0.06 |  |
| Day 1 or Day 2: Low-flow oxygen | 0.06 | Day 1 or Day 2: Low-flow oxygen | 0.06 | Non–COVID-19 infection | 0.06 | Day 1 or Day 2: Low-flow oxygen | 0.06 |  |
| Non–COVID-19 infection | 0.05 | Non–COVID-19 infection | 0.06 | Day 1 or Day 2: Low-flow oxygen | 0.05 | Non–COVID-19 infection | 0.06 |  |
| Day 1 or Day 2: High-flow oxygen | 0.05 | Day 1 or Day 2: High-flow oxygen | 0.06 | Heart failure | 0.05 | Day 1 or Day 2: High-flow oxygen | 0.05 |  |
| Index date: January 2021 | 0.05 | Chest pain | 0.05 | Index date: January 2021 | 0.05 | Chest pain | 0.05 |  |
| Chest pain | 0.05 | Heart failure | 0.05 | Day 1 or Day 2: High-flow oxygen | 0.05 | Index date: January 2021 | 0.05 |  |
| Heart failure | 0.05 | Index date: January 2021 | 0.05 | Chest pain | 0.05 | Smoking | 0.05 |  |
| Smoking | 0.05 | Smoking | 0.04 | Smoking | 0.04 | Heart failure | 0.05 |  |
| Genitourinary condition | 0.04 | Index date: April 2021 | 0.04 | Renal disease (acute) | 0.04 | Age | 0.04 |  |
| Renal disease (acute) | 0.04 | Renal disease (acute) | 0.04 | Genitourinary condition | 0.04 | Genitourinary condition | 0.04 |  |
| Blood condition | 0.04 | Cerebrovascular disease | 0.04 | Day 1 or Day 2: Anticoagulant use | 0.04 | Blood condition | 0.04 |  |
| Index date: April 2021 | 0.04 | Genitourinary condition | 0.04 | Cerebrovascular disease | 0.04 | Index date: April 2021 | 0.04 |  |
| Age | 0.04 | Other forms of heart disease | 0.04 | Other forms of heart disease | 0.04 | Renal disease (acute) | 0.04 |  |
| Day 1 or Day 2: Convalescent plasma use | 0.04 | Blood condition | 0.04 | Index date: April 2021 | 0.04 | Other forms of heart disease | 0.04 |  |
| Cerebrovascular disease | 0.04 | Age | 0.04 | Blood condition | 0.04 | Day 1 or Day 2: Convalescent plasma use | 0.04 |  |
| Other forms of heart disease | 0.04 | Abnormal blood pressure (hypo) | 0.04 | Ischemic heart disease | 0.04 | Cerebrovascular disease | 0.04 |  |
| Ischemic heart disease | 0.04 | Ischemic heart disease | 0.04 | Day 1 or Day 2: Convalescent plasma use | 0.04 | Day 1 or Day 2: Anticoagulant use | 0.04 |  |
| Day 1 or Day 2: Anticoagulant use | 0.04 | Thromboembolic disease | 0.04 | Thromboembolic disease | 0.04 | Headache | 0.04 |  |
| Headache | 0.04 | Day 1 or Day 2: Anticoagulant use | 0.04 | Age | 0.04 | Ischemic heart disease | 0.04 |  |
| Day 1 or Day 2: Immunomodulator use | 0.04 | Behavioral condition | 0.04 | Headache | 0.04 | Thromboembolic disease | 0.04 |  |
| Thromboembolic disease | 0.03 | Day 1 or Day 2: Convalescent plasma use | 0.04 | Day 1 or Day 2: Immunomodulator use | 0.04 | Region: South | 0.04 |  |
| Behavioral condition | 0.03 | Headache | 0.03 | Diseases of arteries, arterioles, and capillaries | 0.03 | Day 1 or Day 2: Immunomodulator use | 0.03 |  |
| Abnormal blood pressure (hypo) | 0.03 | Day 1 or Day 2: Immunomodulator use | 0.03 | Abnormal blood pressure (hypo) | 0.03 | Behavioral condition | 0.03 |  |
| Region: South | 0.03 | Day 1 or Day 2: ICU admission | 0.03 | Conductive disorders, dysrhythmias | 0.03 | Abnormal blood pressure (hypo) | 0.03 |  |
| Day 1 or Day 2: ICU admission | 0.03 | Diseases of arteries, arterioles, and capillaries | 0.03 | Region: South | 0.03 | Diseases of arteries, arterioles, and capillaries | 0.03 |  |
| Diseases of arteries, arterioles, and capillaries | 0.03 | Conductive disorders, dysrhythmias | 0.03 | Region: Northeast | 0.03 | Day 1 or Day 2: ICU admission | 0.03 |  |
| Region: Northeast | 0.03 | Dyspnea/breathlessness | 0.03 | Behavioral condition | 0.03 | Region: Northeast | 0.03 |  |
| Conductive disorders, dysrhythmias | 0.03 | Cough | 0.03 | Digestive condition | 0.03 | Dyspnea/breathlessness | 0.03 |  |
| Digestive condition | 0.03 | Region: South | 0.03 | Day 1 or Day 2: ICU admission | 0.03 | Conductive disorders, dysrhythmias | 0.03 |  |
| Dyspnea/breathlessness | 0.03 | Digestive condition | 0.03 | Dyspnea/breathlessness | 0.03 | Digestive condition | 0.03 |  |
| Cough | 0.03 | Region: Northeast | 0.03 | Cough | 0.03 | Index date: November 2020 | 0.03 |  |
| Index date: November 2020 | 0.02 | Factors influencing health status | 0.03 | Circulatory diseases | 0.03 | Circulatory diseases | 0.02 |  |
| Circulatory diseases | 0.02 | Index date: November 2020 | 0.03 | Index date: November 2020 | 0.03 | Cough | 0.02 |  |
| Other signs/symptoms | 0.02 | Fatigue | 0.02 | Fatigue | 0.02 | Other signs/symptoms | 0.02 |  |
| Fatigue | 0.02 | Circulatory diseases | 0.02 | Other signs/symptoms | 0.02 | Obesity | 0.02 |  |
| Factors influencing health status | 0.02 | Other signs/symptoms | 0.02 | Index date: August 2020 | 0.02 | Fatigue | 0.02 |  |
| Obesity | 0.02 | Obesity | 0.02 | Factors influencing health status | 0.02 | Other ischemic heart disease | 0.02 |  |
| Other ischemic heart disease | 0.02 | Other ischemic heart disease | 0.02 | Other ischemic heart disease | 0.02 | Diseases of veins, lymphatic vessels, and lymph nodes, not elsewhere classified | 0.02 |  |
| Neurologic condition | 0.02 | Index date: August 2020 | 0.02 | Obesity | 0.02 | Factors influencing health status | 0.02 |  |
| Endocrine condition | 0.02 | Neurologic condition | 0.02 | Neurologic condition | 0.02 | Endocrine condition | 0.02 |  |
| Diseases of veins, lymphatic vessels, and lymph nodes, not elsewhere classified | 0.02 | Diarrhea | 0.02 | Smell disturbance/anosmia | 0.02 | Index date: August 2020 | 0.02 |  |
| Index date: August 2020 | 0.02 | Endocrine condition | 0.02 | Index date: August 2021 | 0.02 | Smell disturbance/anosmia | 0.02 |  |
| Smell disturbance/anosmia | 0.02 | Smell disturbance/anosmia | 0.02 | Index date: September 2021 | 0.02 | Neurologic condition | 0.02 |  |
| Index date: September 2021 | 0.02 | Index date: September 2021 | 0.02 | Diseases of veins, lymphatic vessels, and lymph nodes, not elsewhere classified | 0.02 | Index date: September 2021 | 0.02 |  |
| Other and unspecified disorders of the circulatory system | 0.02 | Other and unspecified disorders of the circulatory system | 0.02 | Endocrine condition | 0.02 | Diarrhea | 0.02 |  |
| Diarrhea | 0.02 | Day 1 or Day 2: Protease inhibitor use | 0.02 | Region: West | 0.02 | Index date: August 2021 | 0.02 |  |
| Day 1 or Day 2: Protease inhibitor use | 0.02 | Neoplasms | 0.02 | Other and unspecified disorders of the circulatory system | 0.02 | Index date: February 2021 | 0.02 |  |
| Index date: August 2021 | 0.02 | Region: West | 0.02 | Diabetes | 0.01 | Day 1 or Day 2: Protease inhibitor use | 0.01 |  |
| Neoplasms | 0.02 | Diseases of veins, lymphatic vessels, and lymph nodes, not elsewhere classified | 0.01 | Day 1 or Day 2: Protease inhibitor use | 0.01 | Neoplasms | 0.01 |  |
| Region: West | 0.01 | Index date: August 2021 | 0.01 | Joint pain/arthralgia | 0.01 | Other and unspecified disorders of the circulatory system | 0.01 |  |
| Joint pain/arthralgia | 0.01 | Index date: October 2020 | 0.01 | Diarrhea | 0.01 | Region: West | 0.01 |  |
| Diabetes | 0.01 | Diabetes | 0.01 | Abnormal blood pressure (hyper) | 0.01 | Index date: October 2020 | 0.01 |  |
| Index date: October 2020 | 0.01 | Index date: February 2021 | 0.01 | Neoplasms | 0.01 | Joint pain/arthralgia | 0.01 |  |
| Index date: February 2021 | 0.01 | Joint pain/arthralgia | 0.01 | Index date: October 2020 | 0.01 | Diabetes | 0.01 |  |
| Abnormal blood pressure (hyper) | 0.01 | Region: Midwest | 0.01 | Index date: February 2021 | 0.01 | Region: Midwest | 0.01 |  |
| Dysautonomia | 0.01 | Malformations | 0.01 | Dysautonomia | 0.01 | Dysautonomia | 0.01 |  |
| Region: Midwest | 0.01 | Dysautonomia | 0.01 | Region: Midwest | 0.01 | Evidence of organ transplant | 0.01 |  |
| Malformations | 0.01 | Abnormal blood pressure (hyper) | 0.01 | Index date: May 2021 | 0.01 | Respiratory condition | 0.01 |  |
| Evidence of organ transplant | 0.01 | Diseases of pulmonary circulation | 0.01 | Renal disease (chronic) | 0.01 | Abnormal blood pressure (hyper) | 0.01 |  |
| Respiratory condition | 0.01 | Respiratory condition | 0.01 | Malformations | 0.01 | Malformations | 0.01 |  |
| Musculoskeletal/connective tissue conditions | 0.01 | Evidence of organ transplant | 0.01 | Respiratory condition | 0.01 | Muscle pain/myalgia | 0.01 |  |
| Renal disease (chronic) | 0.01 | Renal disease (chronic) | 0.01 | Index date: September 2020 | 0.01 | Index date: June 2021 | 0.01 |  |
| Taste disturbance/dysgeusia/ageusia | 0.01 | Day 1 or Day 2: ECMO/invasive mechanical ventilation | 0.01 | Evidence of organ transplant | 0.01 | Diseases of pulmonary circulation | 0.01 |  |
| Muscle pain/myalgia | 0.01 | Musculoskeletal/connective tissue conditions | 0.01 | Musculoskeletal/connective tissue conditions | 0.01 | Taste disturbance/dysgeusia/ageusia | 0.01 |  |
| Index date: June 2021 | 0.01 | Taste disturbance/dysgeusia/ageusia | 0.01 | Day 1 or Day 2: ECMO/invasive mechanical ventilation | 0.01 | Musculoskeletal/connective tissue conditions | 0.01 |  |
| Diseases of pulmonary circulation | 0.01 | Index date: March 2021 | 0.01 | Taste disturbance/dysgeusia/ageusia | 0.01 | Day 1 or Day 2: ECMO/invasive mechanical ventilation | 0.01 |  |
| Day 1 or Day 2: ECMO/invasive mechanical ventilation | 0.00 | Index date: June 2021 | 0.01 | Index date: June 2021 | 0.01 | Index date: September 2020 | 0.01 |  |
| Index date: May 2021 | 0.00 | Index date: May 2021 | 0.01 | Metabolic condition | 0.00 | Index date: May 2021 | 0.00 |  |
| Index date: September 2020 | 0.00 | Muscle pain/myalgia | 0.01 | Rheumatic fever | 0.00 | Renal disease (chronic) | 0.00 |  |
| Index date: March 2021 | 0.00 | Rheumatic fever | 0.00 | Sex: Female | 0.00 | Index date: March 2021 | 0.00 |  |
| Sex: Female | 0.00 | Index date: July 2021 | 0.00 | Index date: March 2021 | 0.00 | Sex: Female | 0.00 |  |
| Index date: July 2021 | 0.00 | Index date: September 2020 | 0.00 | Muscle pain/myalgia | 0.00 | Rheumatic fever | 0.00 |  |
| Rheumatic fever | 0.00 | Index date: October 2021 | 0.00 | Index date: July 2021 | 0.00 | Index date: July 2021 | 0.00 |  |
| Index date: October 2021 | 0.00 | Metabolic condition | 0.00 | Index date: October 2021 | 0.00 | Index date: October 2021 | 0.00 |  |
| Metabolic condition | 0.00 | Sex: Female | 0.00 | Diseases of pulmonary circulation | 0.00 | Metabolic condition | 0.00 |  |
|  | | | | | | | | |
| **Fatigue** | | **Dyspnea/breathlessness** | | **Dysautonomia** | | **Diarrhea** | |  |
| **Variable** | **SMD** | **Variable** | **SMD** | **Variable** | **SMD** |  | **SMD** |  |
| Day 1 or Day 2: Corticosteroid use | 0.24 | Day 1 or Day 2: Corticosteroid use | 0.24 | Day 1 or Day 2: Corticosteroid use | 0.23 | Day 1 or Day 2: Corticosteroid use | 0.23 |  |
| Index date: May 2020 | 0.19 | Index date: May 2020 | 0.19 | Index date: May 2020 | 0.19 | Index date: May 2020 | 0.19 |  |
| Index date: June 2020 | 0.10 | Previous COVID-19 infection | 0.10 | Index date: June 2020 | 0.10 | Index date: June 2020 | 0.10 |  |
| Previous COVID-19 infection | 0.10 | Index date: June 2020 | 0.10 | Previous COVID-19 infection | 0.10 | Previous COVID-19 infection | 0.10 |  |
| Mood/neuropsychiatric features | 0.09 | Mood/neuropsychiatric features | 0.10 | Mood/neuropsychiatric features | 0.09 | Mood/neuropsychiatric features | 0.09 |  |
| Index date: July 2020 | 0.08 | Index date: July 2020 | 0.08 | Index date: July 2020 | 0.09 | Index date: July 2020 | 0.09 |  |
| Neuropsychiatric features | 0.08 | Neuropsychiatric features | 0.07 | Neuropsychiatric features | 0.08 | Neuropsychiatric features | 0.08 |  |
| Cognitive dysfunction | 0.07 | Index date: December 2020 | 0.07 | Index date: December 2020 | 0.07 | Index date: December 2020 | 0.07 |  |
| Index date: December 2020 | 0.07 | Cognitive dysfunction | 0.06 | Cognitive dysfunction | 0.06 | Cognitive dysfunction | 0.06 |  |
| Non–COVID-19 infection | 0.06 | Non–COVID-19 infection | 0.06 | Day 1 or Day 2: Low-flow oxygen | 0.06 | Day 1 or Day 2: Low-flow oxygen | 0.06 |  |
| Heart failure | 0.05 | Day 1 or Day 2: Low-flow oxygen | 0.06 | Non–COVID-19 infection | 0.06 | Non–COVID-19 infection | 0.05 |  |
| Day 1 or Day 2: High-flow oxygen | 0.05 | Index date: January 2021 | 0.05 | Day 1 or Day 2: High-flow oxygen | 0.05 | Day 1 or Day 2: High-flow oxygen | 0.05 |  |
| Day 1 or Day 2: Low-flow oxygen | 0.05 | Day 1 or Day 2: High-flow oxygen | 0.05 | Index date: January 2021 | 0.05 | Chest pain | 0.05 |  |
| Index date: January 2021 | 0.05 | Heart failure | 0.05 | Chest pain | 0.05 | Index date: January 2021 | 0.05 |  |
| Chest pain | 0.05 | Chest pain | 0.05 | Heart failure | 0.05 | Heart failure | 0.05 |  |
| Blood condition | 0.05 | Renal disease (acute) | 0.05 | Smoking | 0.05 | Smoking | 0.05 |  |
| Other forms of heart disease | 0.05 | Smoking | 0.04 | Renal disease (acute) | 0.04 | Genitourinary condition | 0.04 |  |
| Renal disease (acute) | 0.04 | Index date: April 2021 | 0.04 | Genitourinary condition | 0.04 | Renal disease (acute) | 0.04 |  |
| Smoking | 0.04 | Blood condition | 0.04 | Blood condition | 0.04 | Blood condition | 0.04 |  |
| Genitourinary condition | 0.04 | Genitourinary condition | 0.04 | Index date: April 2021 | 0.04 | Index date: April 2021 | 0.04 |  |
| Thromboembolic disease | 0.04 | Day 1 or Day 2: Convalescent plasma use | 0.04 | Age | 0.04 | Cerebrovascular disease | 0.04 |  |
| Day 1 or Day 2: Anticoagulant use | 0.04 | Headache | 0.04 | Day 1 or Day 2: Convalescent plasma use | 0.04 | Day 1 or Day 2: Convalescent plasma use | 0.04 |  |
| Day 1 or Day 2: Convalescent plasma use | 0.04 | Thromboembolic disease | 0.04 | Cerebrovascular disease | 0.04 | Age | 0.04 |  |
| Cerebrovascular disease | 0.04 | Age | 0.04 | Other forms of heart disease | 0.04 | Headache | 0.04 |  |
| Index date: April 2021 | 0.04 | Cerebrovascular disease | 0.04 | Ischemic heart disease | 0.04 | Thromboembolic disease | 0.04 |  |
| Headache | 0.04 | Other forms of heart disease | 0.04 | Day 1 or Day 2: Anticoagulant use | 0.04 | Day 1 or Day 2: Anticoagulant use | 0.04 |  |
| Diseases of arteries, arterioles, and capillaries | 0.04 | Behavioral condition | 0.03 | Headache | 0.04 | Day 1 or Day 2: Immunomodulator use | 0.04 |  |
| Ischemic heart disease | 0.04 | Abnormal blood pressure (hypo) | 0.03 | Day 1 or Day 2: Immunomodulator use | 0.04 | Other forms of heart disease | 0.04 |  |
| Day 1 or Day 2: Immunomodulator use | 0.04 | Day 1 or Day 2: Immunomodulator use | 0.03 | Thromboembolic disease | 0.03 | Behavioral condition | 0.04 |  |
| Behavioral condition | 0.04 | Region: Northeast | 0.03 | Behavioral condition | 0.03 | Ischemic heart disease | 0.03 |  |
| Age | 0.04 | Conductive disorders, dysrhythmias | 0.03 | Abnormal blood pressure (hypo) | 0.03 | Abnormal blood pressure (hypo) | 0.03 |  |
| Region: South | 0.04 | Fatigue | 0.03 | Region: South | 0.03 | Day 1 or Day 2: ICU admission | 0.03 |  |
| Abnormal blood pressure (hypo) | 0.03 | Digestive condition | 0.03 | Day 1 or Day 2: ICU admission | 0.03 | Region: South | 0.03 |  |
| Conductive disorders, dysrhythmias | 0.03 | Region: South | 0.03 | Diseases of arteries, arterioles, and capillaries | 0.03 | Region: Northeast | 0.03 |  |
| Region: Northeast | 0.03 | Diseases of arteries, arterioles, and capillaries | 0.03 | Region: Northeast | 0.03 | Digestive condition | 0.03 |  |
| Cough | 0.03 | Day 1 or Day 2: Anticoagulant use | 0.03 | Conductive disorders, dysrhythmias | 0.03 | Conductive disorders, dysrhythmias | 0.03 |  |
| Digestive condition | 0.03 | Ischemic heart disease | 0.03 | Digestive condition | 0.03 | Diseases of arteries, arterioles, and capillaries | 0.03 |  |
| Index date: November 2020 | 0.03 | Day 1 or Day 2: ICU admission | 0.03 | Dyspnea/breathlessness | 0.03 | Dyspnea/breathlessness | 0.03 |  |
| Fatigue | 0.03 | Cough | 0.03 | Cough | 0.03 | Index date: November 2020 | 0.03 |  |
| Day 1 or Day 2: ICU admission | 0.03 | Dyspnea/breathlessness | 0.03 | Circulatory diseases | 0.02 | Cough | 0.03 |  |
| Dyspnea/breathlessness | 0.03 | Index date: August 2020 | 0.03 | Index date: November 2020 | 0.02 | Fatigue | 0.02 |  |
| Circulatory diseases | 0.03 | Circulatory diseases | 0.03 | Other signs/symptoms | 0.02 | Factors influencing health status | 0.02 |  |
| Other ischemic heart disease | 0.03 | Index date: November 2020 | 0.03 | Fatigue | 0.02 | Other signs/symptoms | 0.02 |  |
| Other signs/symptoms | 0.03 | Obesity | 0.02 | Factors influencing health status | 0.02 | Circulatory diseases | 0.02 |  |
| Factors influencing health status | 0.03 | Factors influencing health status | 0.02 | Other ischemic heart disease | 0.02 | Obesity | 0.02 |  |
| Index date: August 2020 | 0.02 | Diarrhea | 0.02 | Obesity | 0.02 | Diseases of veins, lymphatic vessels, and lymph nodes, not elsewhere classified | 0.02 |  |
| Neurologic condition | 0.02 | Other ischemic heart disease | 0.02 | Neurologic condition | 0.02 | Other ischemic heart disease | 0.02 |  |
| Diseases of veins, lymphatic vessels, and lymph nodes, not elsewhere classified | 0.02 | Other signs/symptoms | 0.02 | Endocrine condition | 0.02 | Index date: August 2020 | 0.02 |  |
| Other and unspecified disorders of the circulatory system | 0.02 | Other and unspecified disorders of the circulatory system | 0.02 | Diseases of veins, lymphatic vessels, and lymph nodes, not elsewhere classified | 0.02 | Smell disturbance/anosmia | 0.02 |  |
| Diarrhea | 0.02 | Neurologic condition | 0.02 | Smell disturbance/anosmia | 0.02 | Neurologic condition | 0.02 |  |
| Obesity | 0.02 | Region: West | 0.02 | Index date: August 2020 | 0.02 | Index date: September 2021 | 0.02 |  |
| Neoplasms | 0.02 | Neoplasms | 0.02 | Index date: September 2021 | 0.02 | Diarrhea | 0.02 |  |
| Diabetes | 0.02 | Index date: September 2021 | 0.02 | Other and unspecified disorders of the circulatory system | 0.02 | Endocrine condition | 0.02 |  |
| Index date: August 2021 | 0.02 | Diseases of veins, lymphatic vessels, and lymph nodes, not elsewhere classified | 0.02 | Day 1 or Day 2: Protease inhibitor use | 0.02 | Index date: August 2021 | 0.02 |  |
| Endocrine condition | 0.02 | Index date: August 2021 | 0.02 | Diarrhea | 0.02 | Day 1 or Day 2: Protease inhibitor use | 0.02 |  |
| Smell disturbance/anosmia | 0.02 | Endocrine condition | 0.02 | Index date: August 2021 | 0.02 | Other and unspecified disorders of the circulatory system | 0.02 |  |
| Index date: September 2021 | 0.02 | Diabetes | 0.02 | Neoplasms | 0.02 | Neoplasms | 0.02 |  |
| Day 1 or Day 2: Protease inhibitor use | 0.02 | Day 1 or Day 2: Protease inhibitor use | 0.01 | Region: West | 0.01 | Region: West | 0.02 |  |
| Region: West | 0.01 | Smell disturbance/anosmia | 0.01 | Joint pain/arthralgia | 0.01 | Index date: October 2020 | 0.01 |  |
| Renal disease (chronic) | 0.01 | Index date: October 2020 | 0.01 | Index date: October 2020 | 0.01 | Joint pain/arthralgia | 0.01 |  |
| Abnormal blood pressure (hyper) | 0.01 | Region: Midwest | 0.01 | Diabetes | 0.01 | Diabetes | 0.01 |  |
| Joint pain/arthralgia | 0.01 | Index date: February 2021 | 0.01 | Index date: February 2021 | 0.01 | Abnormal blood pressure (hyper) | 0.01 |  |
| Musculoskeletal/connective tissue conditions | 0.01 | Musculoskeletal/connective tissue conditions | 0.01 | Abnormal blood pressure (hyper) | 0.01 | Dysautonomia | 0.01 |  |
| Index date: October 2020 | 0.01 | Dysautonomia | 0.01 | Dysautonomia | 0.01 | Index date: February 2021 | 0.01 |  |
| Index date: February 2021 | 0.01 | Index date: June 2021 | 0.01 | Region: Midwest | 0.01 | Region: Midwest | 0.01 |  |
| Index date: September 2020 | 0.01 | Index date: September 2020 | 0.01 | Malformations | 0.01 | Evidence of organ transplant | 0.01 |  |
| Region: Midwest | 0.01 | Joint pain/arthralgia | 0.01 | Evidence of organ transplant | 0.01 | Musculoskeletal/connective tissue conditions | 0.01 |  |
| Dysautonomia | 0.01 | Renal disease (chronic) | 0.01 | Respiratory condition | 0.01 | Malformations | 0.01 |  |
| Index date: June 2021 | 0.01 | Abnormal blood pressure (hyper) | 0.01 | Musculoskeletal/connective tissue conditions | 0.01 | Respiratory condition | 0.01 |  |
| Respiratory condition | 0.01 | Muscle pain/myalgia | 0.01 | Renal disease (chronic) | 0.01 | Index date: June 2021 | 0.01 |  |
| Index date: May 2021 | 0.01 | Respiratory condition | 0.01 | Taste disturbance/dysgeusia/ageusia | 0.01 | Muscle pain/myalgia | 0.01 |  |
| Malformations | 0.01 | Index date: March 2021 | 0.01 | Index date: June 2021 | 0.01 | Taste disturbance/dysgeusia/ageusia | 0.01 |  |
| Muscle pain/myalgia | 0.01 | Taste disturbance/dysgeusia/ageusia | 0.01 | Muscle pain/myalgia | 0.01 | Renal disease (chronic) | 0.01 |  |
| Diseases of pulmonary circulation | 0.01 | Diseases of pulmonary circulation | 0.00 | Diseases of pulmonary circulation | 0.01 | Index date: September 2020 | 0.00 |  |
| Taste disturbance/dysgeusia/ageusia | 0.00 | Malformations | 0.00 | Day 1 or Day 2: ECMO/invasive mechanical ventilation | 0.00 | Diseases of pulmonary circulation | 0.00 |  |
| Index date: March 2021 | 0.00 | Index date: July 2021 | 0.00 | Index date: May 2021 | 0.00 | Index date: March 2021 | 0.00 |  |
| Evidence of organ transplant | 0.00 | Index date: May 2021 | 0.00 | Index date: September 2020 | 0.00 | Index date: May 2021 | 0.00 |  |
| Index date: July 2021 | 0.00 | Evidence of organ transplant | 0.00 | Index date: March 2021 | 0.00 | Sex: Female | 0.00 |  |
| Sex: Female | 0.00 | Rheumatic fever | 0.00 | Index date: July 2021 | 0.00 | Index date: July 2021 | 0.00 |  |
| Metabolic condition | 0.00 | Sex: Female | 0.00 | Sex: Female | 0.00 | Rheumatic fever | 0.00 |  |
| Index date: October 2021 | 0.00 | Day 1 or Day 2: ECMO/invasive mechanical ventilation | 0.00 | Rheumatic fever | 0.00 | Index date: October 2021 | 0.00 |  |
| Rheumatic fever | 0.00 | Metabolic condition | 0.00 | Index date: October 2021 | 0.00 | Day 1 or Day 2: ECMO/invasive mechanical ventilation | 0.00 |  |
| Day 1 or Day 2: ECMO/invasive mechanical ventilation | 0.00 | Index date: October 2021 | 0.00 | Metabolic condition | 0.00 | Metabolic condition | 0.00 |  |
|  | | | | | | | | |
| **Cerebrovascular disease** | | **Cough** | | **Cognitive dysfunction** | | **Chest pain** | |  |
| **Variable** | **SMD** | **Variable** | **SMD** | **Variable** | **SMD** | **Variable** | **SMD** |  |
| Day 1 or Day 2: Corticosteroid use | 0.23 | Day 1 or Day 2: Corticosteroid use | 0.23 | Day 1 or Day 2: Corticosteroid use | 0.23 | Day 1 or Day 2: Corticosteroid use | 0.23 |  |
| Index date: May 2020 | 0.19 | Index date: May 2020 | 0.19 | Index date: May 2020 | 0.19 | Index date: May 2020 | 0.19 |  |
| Previous COVID-19 infection | 0.10 | Previous COVID-19 infection | 0.10 | Previous COVID-19 infection | 0.10 | Index date: June 2020 | 0.10 |  |
| Index date: June 2020 | 0.10 | Index date: June 2020 | 0.10 | Index date: June 2020 | 0.10 | Previous COVID-19 infection | 0.10 |  |
| Mood/neuropsychiatric features | 0.09 | Mood/neuropsychiatric features | 0.09 | Mood/neuropsychiatric features | 0.10 | Index date: July 2020 | 0.09 |  |
| Index date: July 2020 | 0.09 | Index date: July 2020 | 0.09 | Index date: July 2020 | 0.09 | Mood/neuropsychiatric features | 0.09 |  |
| Neuropsychiatric features | 0.07 | Neuropsychiatric features | 0.08 | Neuropsychiatric features | 0.08 | Neuropsychiatric features | 0.07 |  |
| Index date: December 2020 | 0.07 | Index date: December 2020 | 0.07 | Cognitive dysfunction | 0.07 | Index date: December 2020 | 0.07 |  |
| Cognitive dysfunction | 0.06 | Cognitive dysfunction | 0.07 | Index date: December 2020 | 0.06 | Cognitive dysfunction | 0.06 |  |
| Non–COVID-19 infection | 0.06 | Day 1 or Day 2: Low-flow oxygen | 0.06 | Non–COVID-19 infection | 0.06 | Non–COVID-19 infection | 0.05 |  |
| Day 1 or Day 2: Low-flow oxygen | 0.06 | Non–COVID-19 infection | 0.06 | Index date: January 2021 | 0.05 | Chest pain | 0.05 |  |
| Heart failure | 0.05 | Day 1 or Day 2: High-flow oxygen | 0.05 | Heart failure | 0.05 | Day 1 or Day 2: Low-flow oxygen | 0.05 |  |
| Index date: January 2021 | 0.05 | Chest pain | 0.05 | Day 1 or Day 2: Low-flow oxygen | 0.05 | Day 1 or Day 2: High-flow oxygen | 0.05 |  |
| Day 1 or Day 2: High-flow oxygen | 0.05 | Index date: January 2021 | 0.05 | Day 1 or Day 2: High-flow oxygen | 0.05 | Index date: January 2021 | 0.05 |  |
| Index date: April 2021 | 0.05 | Heart failure | 0.05 | Chest pain | 0.05 | Heart failure | 0.05 |  |
| Chest pain | 0.05 | Smoking | 0.05 | Blood condition | 0.05 | Smoking | 0.05 |  |
| Blood condition | 0.04 | Blood condition | 0.04 | Cerebrovascular disease | 0.04 | Blood condition | 0.04 |  |
| Genitourinary condition | 0.04 | Renal disease (acute) | 0.04 | Renal disease (acute) | 0.04 | Renal disease (acute) | 0.04 |  |
| Cerebrovascular disease | 0.04 | Index date: April 2021 | 0.04 | Index date: April 2021 | 0.04 | Genitourinary condition | 0.04 |  |
| Smoking | 0.04 | Genitourinary condition | 0.04 | Other forms of heart disease | 0.04 | Cerebrovascular disease | 0.04 |  |
| Day 1 or Day 2: Anticoagulant use | 0.04 | Age | 0.04 | Genitourinary condition | 0.04 | Other forms of heart disease | 0.04 |  |
| Abnormal blood pressure (hypo) | 0.04 | Day 1 or Day 2: Convalescent plasma use | 0.04 | Smoking | 0.04 | Ischemic heart disease | 0.04 |  |
| Renal disease (acute) | 0.04 | Other forms of heart disease | 0.04 | Day 1 or Day 2: Convalescent plasma use | 0.04 | Index date: April 2021 | 0.04 |  |
| Thromboembolic disease | 0.04 | Ischemic heart disease | 0.04 | Day 1 or Day 2: Anticoagulant use | 0.04 | Day 1 or Day 2: Convalescent plasma use | 0.04 |  |
| Day 1 or Day 2: Convalescent plasma use | 0.04 | Headache | 0.04 | Headache | 0.04 | Day 1 or Day 2: Anticoagulant use | 0.04 |  |
| Other forms of heart disease | 0.04 | Cerebrovascular disease | 0.04 | Diseases of arteries, arterioles, and capillaries | 0.04 | Thromboembolic disease | 0.04 |  |
| Age | 0.04 | Day 1 or Day 2: Immunomodulator use | 0.04 | Abnormal blood pressure (hypo) | 0.04 | Age | 0.04 |  |
| Region: South | 0.04 | Region: South | 0.03 | Thromboembolic disease | 0.04 | Day 1 or Day 2: Immunomodulator use | 0.04 |  |
| Behavioral condition | 0.03 | Day 1 or Day 2: Anticoagulant use | 0.03 | Behavioral condition | 0.04 | Abnormal blood pressure (hypo) | 0.04 |  |
| Headache | 0.03 | Abnormal blood pressure (hypo) | 0.03 | Day 1 or Day 2: Immunomodulator use | 0.04 | Behavioral condition | 0.03 |  |
| Diseases of arteries, arterioles, and capillaries | 0.03 | Thromboembolic disease | 0.03 | Age | 0.03 | Headache | 0.03 |  |
| Day 1 or Day 2: Immunomodulator use | 0.03 | Behavioral condition | 0.03 | Conductive disorders, dysrhythmias | 0.03 | Conductive disorders, dysrhythmias | 0.03 |  |
| Region: Northeast | 0.03 | Conductive disorders, dysrhythmias | 0.03 | Region: Northeast | 0.03 | Diseases of arteries, arterioles, and capillaries | 0.03 |  |
| Digestive condition | 0.03 | Cough | 0.03 | Day 1 or Day 2: ICU admission | 0.03 | Dyspnea/breathlessness | 0.03 |  |
| Index date: November 2020 | 0.03 | Diseases of arteries, arterioles, and capillaries | 0.03 | Circulatory diseases | 0.03 | Region: Northeast | 0.03 |  |
| Ischemic heart disease | 0.03 | Day 1 or Day 2: ICU admission | 0.03 | Region: South | 0.03 | Digestive condition | 0.03 |  |
| Circulatory diseases | 0.03 | Digestive condition | 0.03 | Cough | 0.03 | Region: South | 0.03 |  |
| Day 1 or Day 2: ICU admission | 0.03 | Region: Northeast | 0.03 | Ischemic heart disease | 0.03 | Day 1 or Day 2: ICU admission | 0.03 |  |
| Other signs/symptoms | 0.03 | Index date: November 2020 | 0.03 | Fatigue | 0.03 | Cough | 0.03 |  |
| Conductive disorders, dysrhythmias | 0.03 | Dyspnea/breathlessness | 0.03 | Dyspnea/breathlessness | 0.03 | Index date: November 2020 | 0.03 |  |
| Dyspnea/breathlessness | 0.02 | Circulatory diseases | 0.02 | Digestive condition | 0.03 | Fatigue | 0.02 |  |
| Diseases of veins, lymphatic vessels, and lymph nodes, not elsewhere classified | 0.02 | Fatigue | 0.02 | Diseases of veins, lymphatic vessels, and lymph nodes, not elsewhere classified | 0.03 | Other ischemic heart disease | 0.02 |  |
| Fatigue | 0.02 | Other ischemic heart disease | 0.02 | Index date: November 2020 | 0.03 | Factors influencing health status | 0.02 |  |
| Factors influencing health status | 0.02 | Other signs/symptoms | 0.02 | Other signs/symptoms | 0.02 | Obesity | 0.02 |  |
| Other ischemic heart disease | 0.02 | Index date: August 2020 | 0.02 | Other ischemic heart disease | 0.02 | Circulatory diseases | 0.02 |  |
| Index date: August 2020 | 0.02 | Factors influencing health status | 0.02 | Factors influencing health status | 0.02 | Index date: August 2020 | 0.02 |  |
| Obesity | 0.02 | Obesity | 0.02 | Index date: August 2020 | 0.02 | Other signs/symptoms | 0.02 |  |
| Neurologic condition | 0.02 | Neurologic condition | 0.02 | Neurologic condition | 0.02 | Other and unspecified disorders of the circulatory system | 0.02 |  |
| Cough | 0.02 | Endocrine condition | 0.02 | Obesity | 0.02 | Endocrine condition | 0.02 |  |
| Diarrhea | 0.02 | Diarrhea | 0.02 | Neoplasms | 0.02 | Neurologic condition | 0.02 |  |
| Smell disturbance/anosmia | 0.02 | Other and unspecified disorders of the circulatory system | 0.02 | Index date: August 2021 | 0.02 | Index date: February 2021 | 0.02 |  |
| Diabetes | 0.02 | Diseases of veins, lymphatic vessels, and lymph nodes, not elsewhere classified | 0.02 | Other and unspecified disorders of the circulatory system | 0.02 | Index date: August 2021 | 0.02 |  |
| Index date: August 2021 | 0.02 | Index date: September 2021 | 0.02 | Endocrine condition | 0.02 | Diarrhea | 0.02 |  |
| Day 1 or Day 2: Protease inhibitor use | 0.02 | Smell disturbance/anosmia | 0.02 | Index date: September 2021 | 0.02 | Diseases of veins, lymphatic vessels, and lymph nodes, not elsewhere classified | 0.02 |  |
| Other and unspecified disorders of the circulatory system | 0.02 | Day 1 or Day 2: Protease inhibitor use | 0.02 | Diabetes | 0.02 | Index date: September 2021 | 0.02 |  |
| Index date: September 2021 | 0.02 | Index date: August 2021 | 0.02 | Smell disturbance/anosmia | 0.02 | Neoplasms | 0.02 |  |
| Neoplasms | 0.02 | Neoplasms | 0.02 | Region: West | 0.02 | Diabetes | 0.02 |  |
| Joint pain/arthralgia | 0.01 | Region: West | 0.01 | Index date: February 2021 | 0.02 | Smell disturbance/anosmia | 0.02 |  |
| Index date: February 2021 | 0.01 | Diabetes | 0.01 | Day 1 or Day 2: Protease inhibitor use | 0.01 | Day 1 or Day 2: Protease inhibitor use | 0.01 |  |
| Endocrine condition | 0.01 | Index date: October 2020 | 0.01 | Diarrhea | 0.01 | Region: West | 0.01 |  |
| Malformations | 0.01 | Joint pain/arthralgia | 0.01 | Malformations | 0.01 | Index date: October 2020 | 0.01 |  |
| Region: West | 0.01 | Abnormal blood pressure (hyper) | 0.01 | Index date: October 2020 | 0.01 | Joint pain/arthralgia | 0.01 |  |
| Dysautonomia | 0.01 | Region: Midwest | 0.01 | Abnormal blood pressure (hyper) | 0.01 | Dysautonomia | 0.01 |  |
| Musculoskeletal/connective tissue conditions | 0.01 | Musculoskeletal/connective tissue conditions | 0.01 | Musculoskeletal/connective tissue conditions | 0.01 | Abnormal blood pressure (hyper) | 0.01 |  |
| Abnormal blood pressure (hyper) | 0.01 | Dysautonomia | 0.01 | Region: Midwest | 0.01 | Malformations | 0.01 |  |
| Respiratory condition | 0.01 | Renal disease (chronic) | 0.01 | Joint pain/arthralgia | 0.01 | Renal disease (chronic) | 0.01 |  |
| Index date: October 2020 | 0.01 | Index date: February 2021 | 0.01 | Index date: September 2020 | 0.01 | Respiratory condition | 0.01 |  |
| Region: Midwest | 0.01 | Diseases of pulmonary circulation | 0.01 | Index date: May 2021 | 0.01 | Index date: May 2021 | 0.01 |  |
| Renal disease (chronic) | 0.01 | Evidence of organ transplant | 0.01 | Respiratory condition | 0.01 | Index date: September 2020 | 0.01 |  |
| Index date: September 2020 | 0.01 | Respiratory condition | 0.01 | Dysautonomia | 0.01 | Index date: June 2021 | 0.01 |  |
| Muscle pain/myalgia | 0.01 | Index date: September 2020 | 0.01 | Renal disease (chronic) | 0.01 | Region: Midwest | 0.01 |  |
| Evidence of organ transplant | 0.01 | Taste disturbance/dysgeusia/ageusia | 0.01 | Taste disturbance/dysgeusia/ageusia | 0.01 | Musculoskeletal/connective tissue conditions | 0.01 |  |
| Diseases of pulmonary circulation | 0.01 | Malformations | 0.01 | Index date: March 2021 | 0.01 | Evidence of organ transplant | 0.01 |  |
| Taste disturbance/dysgeusia/ageusia | 0.01 | Index date: June 2021 | 0.01 | Evidence of organ transplant | 0.01 | Taste disturbance/dysgeusia/ageusia | 0.01 |  |
| Index date: March 2021 | 0.00 | Muscle pain/myalgia | 0.01 | Diseases of pulmonary circulation | 0.00 | Diseases of pulmonary circulation | 0.00 |  |
| Index date: July 2021 | 0.00 | Index date: May 2021 | 0.01 | Day 1 or Day 2: ECMO/invasive mechanical ventilation | 0.00 | Day 1 or Day 2: ECMO/invasive mechanical ventilation | 0.00 |  |
| Index date: May 2021 | 0.00 | Day 1 or Day 2: ECMO/invasive mechanical ventilation | 0.00 | Rheumatic fever | 0.00 | Metabolic condition | 0.00 |  |
| Day 1 or Day 2: ECMO/invasive mechanical ventilation | 0.00 | Index date: March 2021 | 0.00 | Sex: Female | 0.00 | Muscle pain/myalgia | 0.00 |  |
| Metabolic condition | 0.00 | Index date: July 2021 | 0.00 | Index date: October 2021 | 0.00 | Index date: March 2021 | 0.00 |  |
| Index date: October 2021 | 0.00 | Metabolic condition | 0.00 | Index date: July 2021 | 0.00 | Sex: Female | 0.00 |  |
| Index date: June 2021 | 0.00 | Index date: October 2021 | 0.00 | Muscle pain/myalgia | 0.00 | Index date: July 2021 | 0.00 |  |
| Rheumatic fever | 0.00 | Rheumatic fever | 0.00 | Index date: June 2021 | 0.00 | Index date: October 2021 | 0.00 |  |
| Sex: Female | 0.00 | Sex: Female | 0.00 | Metabolic condition | 0.00 | Rheumatic fever | 0.00 |  |
|  | | | | | | | | |
| **Thromboembolic disease** | |  |  |  |  |  |  |  |
| **Variable** | **SMD** |  |  |  |  |  |  |  |
| Day 1 or Day 2: Corticosteroid use | 0.23 |  |  |  |  |  |  |  |
| Index date: May 2020 | 0.19 |  |  |  |  |  |  |  |
| Index date: June 2020 | 0.10 |  |  |  |  |  |  |  |
| Previous COVID-19 infection | 0.09 |  |  |  |  |  |  |  |
| Mood/neuropsychiatric features | 0.09 |  |  |  |  |  |  |  |
| Index date: July 2020 | 0.08 |  |  |  |  |  |  |  |
| Neuropsychiatric features | 0.08 |  |  |  |  |  |  |  |
| Index date: December 2020 | 0.07 |  |  |  |  |  |  |  |
| Cognitive dysfunction | 0.06 |  |  |  |  |  |  |  |
| Chest pain | 0.06 |  |  |  |  |  |  |  |
| Non–COVID-19 infection | 0.06 |  |  |  |  |  |  |  |
| Day 1 or Day 2: Low-flow oxygen | 0.05 |  |  |  |  |  |  |  |
| Day 1 or Day 2: High-flow oxygen | 0.05 |  |  |  |  |  |  |  |
| Index date: January 2021 | 0.05 |  |  |  |  |  |  |  |
| Heart failure | 0.05 |  |  |  |  |  |  |  |
| Smoking | 0.05 |  |  |  |  |  |  |  |
| Blood condition | 0.04 |  |  |  |  |  |  |  |
| Renal disease (acute) | 0.04 |  |  |  |  |  |  |  |
| Genitourinary condition | 0.04 |  |  |  |  |  |  |  |
| Anticoagulant | 0.04 |  |  |  |  |  |  |  |
| Thromboembolic | 0.04 |  |  |  |  |  |  |  |
| Cerebrovascular disease | 0.04 |  |  |  |  |  |  |  |
| Other forms of heart disease | 0.04 |  |  |  |  |  |  |  |
| Day 1 or Day 2: Convalescent plasma use | 0.04 |  |  |  |  |  |  |  |
| Index date: April 2021 | 0.04 |  |  |  |  |  |  |  |
| Age | 0.04 |  |  |  |  |  |  |  |
| Ischemic heart disease | 0.04 |  |  |  |  |  |  |  |
| Abnormal blood pressure (hypo) | 0.04 |  |  |  |  |  |  |  |
| Behavioral condition | 0.04 |  |  |  |  |  |  |  |
| Day 1 or Day 2: Immunomodulator use | 0.03 |  |  |  |  |  |  |  |
| Diseases of arteries, arterioles, and capillaries | 0.03 |  |  |  |  |  |  |  |
| Region: South | 0.03 |  |  |  |  |  |  |  |
| Headache | 0.03 |  |  |  |  |  |  |  |
| Conductive disorders, dysrhythmias | 0.03 |  |  |  |  |  |  |  |
| Day 1 or Day 2: ICU admission | 0.03 |  |  |  |  |  |  |  |
| Region: Northeast | 0.03 |  |  |  |  |  |  |  |
| Cough | 0.03 |  |  |  |  |  |  |  |
| Dyspnea/breathlessness | 0.03 |  |  |  |  |  |  |  |
| Digestive condition | 0.03 |  |  |  |  |  |  |  |
| Fatigue | 0.03 |  |  |  |  |  |  |  |
| Factors influencing health status | 0.03 |  |  |  |  |  |  |  |
| Other ischemic heart disease | 0.03 |  |  |  |  |  |  |  |
| Obesity | 0.02 |  |  |  |  |  |  |  |
| Index date: November 2020 | 0.02 |  |  |  |  |  |  |  |
| Circulatory diseases | 0.02 |  |  |  |  |  |  |  |
| Other signs/symptoms | 0.02 |  |  |  |  |  |  |  |
| Neurologic condition | 0.02 |  |  |  |  |  |  |  |
| Diseases of veins, lymphatic vessels, and lymph nodes, not elsewhere classified | 0.02 |  |  |  |  |  |  |  |
| Index date: August 2020 | 0.02 |  |  |  |  |  |  |  |
| Endocrine condition | 0.02 |  |  |  |  |  |  |  |
| Other and unspecified disorders of the circulatory system | 0.02 |  |  |  |  |  |  |  |
| Diarrhea | 0.02 |  |  |  |  |  |  |  |
| Smell disturbance/anosmia | 0.02 |  |  |  |  |  |  |  |
| Index date: September 2021 | 0.02 |  |  |  |  |  |  |  |
| Index date: August 2021 | 0.02 |  |  |  |  |  |  |  |
| Day 1 or Day 2: Protease inhibitor use | 0.02 |  |  |  |  |  |  |  |
| Joint pain/arthralgia | 0.01 |  |  |  |  |  |  |  |
| Diabetes | 0.01 |  |  |  |  |  |  |  |
| Neoplasms | 0.01 |  |  |  |  |  |  |  |
| Index date: February 2021 | 0.01 |  |  |  |  |  |  |  |
| Index date: October 2020 | 0.01 |  |  |  |  |  |  |  |
| Region: West | 0.01 |  |  |  |  |  |  |  |
| Abnormal blood pressure (hyper) | 0.01 |  |  |  |  |  |  |  |
| Malformations | 0.01 |  |  |  |  |  |  |  |
| Respiratory condition | 0.01 |  |  |  |  |  |  |  |
| Dysautonomia | 0.01 |  |  |  |  |  |  |  |
| Musculoskeletal/connective tissue conditions | 0.01 |  |  |  |  |  |  |  |
| Renal disease (chronic) | 0.01 |  |  |  |  |  |  |  |
| Muscle pain/myalgia | 0.01 |  |  |  |  |  |  |  |
| Region: Midwest | 0.01 |  |  |  |  |  |  |  |
| Index date: March 2021 | 0.01 |  |  |  |  |  |  |  |
| Index date: September 2020 | 0.01 |  |  |  |  |  |  |  |
| Index date: June 2021 | 0.01 |  |  |  |  |  |  |  |
| Taste disturbance/dysgeusia/ageusia | 0.01 |  |  |  |  |  |  |  |
| Evidence of organ transplant | 0.00 |  |  |  |  |  |  |  |
| Diseases of pulmonary circulation | 0.00 |  |  |  |  |  |  |  |
| Index date: May 2021 | 0.00 |  |  |  |  |  |  |  |
| Index date: July 2021 | 0.00 |  |  |  |  |  |  |  |
| Rheumatic fever | 0.00 |  |  |  |  |  |  |  |
| Sex: Female | 0.00 |  |  |  |  |  |  |  |
| Index date: October 2021 | 0.00 |  |  |  |  |  |  |  |
| Day 1 or Day 2: ECMO/invasive mechanical ventilation | 0.00 |  |  |  |  |  |  |  |
| Metabolic condition | 0.00 |  |  |  |  |  |  |  |

ECMO, extracorporeal membrane oxygenation; ICU, intensive care unit; SMD, standardized mean difference.

# Supplementary Table 4. Subgroup Definitions

| **Subgroup** | **Definition and code list** |
| --- | --- |
| Immunocompromised^5^ | At least 1 ICD-10 diagnosis code in claims or chargemaster data in the 365 days prior to the index date   - HIV/AIDS: B20-B24 - Hematologic malignancy: C81-C83, C88-C96 - Other immune conditions: D89, D70, D71, D72.0, D72.81, D72.89, D72.9, D75.81, D47.4, D75.89, D75.9, D89.2, D75.89, R76, R83.4-R87.4, R89.4 - Solid malignancy: C00-C07, C11-C19, C22-80, Z85, C7A, C7B, D3A, D00-D49 - Organ transplant: T86, Z94, Z98.85 - Rheumatologic/inflammatory: D86, E85, E85.0, M04, E85.1, E85.3, E85.8, G35, G36, G37.1, G37.3, G37.8, G37.9, G61.0, G61.9, I40, M30, T78.40, J67.9, J84.01, J84.02, J84.09, K50-K52, L93.0, L93.2, M32, L94, M35.8, M35.9, M12.9, M01.X0, M02.10, M11, M05-M14, M46, M31.5, M35.3 |
| Patients with a therapy or condition associated with a moderately-to-severely immunocompromised state based on NIH definition^6^ | - At least 1 claim/record in chargemaster data with an ICD-10 diagnosis code for cancer (solid tumor or hematologic malignancies, as above) AND 1 pharmacy claim with ATC code L01 or L02 OR a procedure code indicating antineoplastic chemotherapy administration (Z51.11) in chargemaster or data within 180 days prior to the index date - At least 1 claim/record in chargemaster data with a diagnosis code/procedure code for solid-organ transplant (as above) any time prior to index date and at least 1 claim/record in chargemaster data with drug codes for 1 of the following drugs within 180 days prior to the index dates: tacrolimus, mycophenolate, prednisone, cyclosporine, everolimus, sirolimus, azathioprine, belatacept, or antithymocyte globulin - At least 1 claim/record in chargemaster data with a procedure code or drug code indicating CAR-T (XW033J7, XW043M7, XW03351, XW033H7, Z92.850) or hematopoietic cell transplant (Z94.84) within 730 days prior to the index date - At least 1 claim/record in chargemaster data with a procedure code or drug code indicating CAR-T or hematopoietic cell transplant (as above) any time prior to index date AND at least 1 claim/record in chargemaster data with drug codes for 1 of the following drugs within 180 days prior to the index date: tacrolimus, mycophenolate, prednisone, cyclosporine, everolimus, sirolimus, azathioprine, belatacept, or antithymocyte globulin - At least 1 claim/record in chargemaster data with a diagnosis code for moderate or severe primary immunodeficiency (D81, D83) at any point prior to the index date - At least 1 claim/record in chargemaster data with a diagnosis code for HIV (as above) AND a diagnosis code for an opportunistic infection (A02, A07.2, A07.3, A15, A17, A18, A19, A31, A32, A40.3, A42, A43, A44, A48.1, A48.2, A81.2, B00, B01, B02, B16, B17, B18, B19, B25, B27, B37, B38, B39, B40, B44, B45, B55, B58, B59, B78, B95.3, G02, J13, K23) within 180 days of the index date OR no claims for antiretroviral medication within the 365 days period to the index date - At least 2 claims for high-dose steroids, defined as claims for IV corticosteroid or for oral corticosteroids (prednisone, methylprednisolone, hydrocortisone, or dexamethasone) with a dose of  ≥20 mg (or prednisone equivalent in mg) within 180 days prior to the index date |

ATC, Anatomical Therapeutic Chemical; CAR-T, chimeric antigen receptor T; ICD-10, *International Classification of Diseases, Tenth Revision*; IV, intravenous; NIH, National Institutes of Health.

# Supplementary Table 5. Long COVID-Associated Outcome ICD-10 Diagnosis Codes

| **Outcome** | **ICD-10 codes** |
| --- | --- |
| Cough | R05 – Cough |
| Chest pain | R07.1 – Chest pain on breathing  R07.2 – Precordial pain  R07.8 – Other chest pain  R07.81 – Pleurodynia  R07.82 – Intercostal pain  R07.89 – Other chest pain  R07.9 – Chest pain, unspecified |
| Cerebrovascular disease | I60-I69 – Stroke  G45 – Transient ischemic attack |
| Cognitive dysfunction | A81.00 – Creutzfeldt-Jakob disease, unspecified  E71.0 – Maple syrup urine disease  E75.2 – Other sphingolipidosis  F01.5 – Vascular dementia  F02.8 – Dementia in other diseases classified elsewhere  F03.9 – Unspecified dementia  F04 – Amnestic disorder due to known physiological condition  F10.26 – Alcohol dependence with alcohol-induced persisting amnestic disorder  F10.27 – Alcohol dependence with alcohol-induced persisting dementia  F10.96 – Alcohol use, unspecified with alcohol-induced persisting amnestic disorder  F10.97 – Alcohol use, unspecified with alcohol-induced persisting dementia  F13.26 – Sedative, hypnotic, or anxiolytic dependence with sedative-, hypnotic-, or anxiolytic-induced persisting amnestic disorder  F13.27 – Sedative, hypnotic, or anxiolytic dependence with sedative-, hypnotic-, or anxiolytic-induced persisting dementia  F13.96 – Sedative, hypnotic, or anxiolytic use, unspecified with sedative-, hypnotic-, or anxiolytic-induced persisting amnestic disorder  F13.97 – Sedative, hypnotic, or anxiolytic use, unspecified with sedative-, hypnotic-, or anxiolytic-induced persisting dementia  F18.27 – Inhalant dependence with inhalant-induced dementia  F18.97 – Inhalant use, unspecified with inhalant-induced persisting dementia  G10 – Huntington’s disease  G20 – Parkinson’s disease  G23.1 – Progressive supranuclear ophthalmoplegia (Steele-Richardson-Olszewski)  G30 – Alzheimer’s disease  G31 – Other degenerative diseases of the nervous system  R41 – Disorientation |
| Dyspnea/breathlessness | R06.02 – Shortness of breath  R06.0 – Breathlessness/dyspnea  R06.2 – Wheezing |
| Diarrhea | R19.7 – Diarrhea, unspecified |
| Dysautonomia | G90.9 – Disorder of the autonomic nervous system, unspecified |
| Fatigue | R53.1 – Weakness  R53.81 – Other malaise  R53.83 – Other fatigue  G93.3 – Postviral fatigue syndrome |
| Headache | R51 – Headache |
| Ischemic heart disease | I24 – Acute coronary disease  I21, I22 – Myocardial infarction  I25.5 – Ischemic cardiomyopathy  I20 – Angina |
| Joint pain/arthralgia | M25.5 – Pain in joint  M25.6 – Stiffness of joint, not elsewhere classified |
| Long COVID | U09.9 – Post COVID-19 condition, unspecified |
| Muscle pain/myalgia | M79.1 – Myalgia  M79.10 – Myalgia, unspecified site  M79.11 – Myalgia of mastication muscle  M79.12 – Myalgia of auxiliary muscles, head, and neck  M79.18 – Myalgia, other site  M79 – Other and unspecified soft-tissue disorders, not elsewhere classified |
| Neuropsychiatric features | F10 – Alcohol-related disorders  F11 – Opioid-related disorders  F12 – Cannabis-related disorders  F13 – Sedative-, hypnotic-, or anxiolytic-related disorders  F14 – Cocaine-related disorders  F15 – Other stimulant–related disorders  F16 – Hallucinogen-related disorders  F17 – Nicotine dependence  F18 – Inhalant-related disorders  F19 – Other psychoactive substance–related disorders  F20 – Schizophrenia  F21 – Schizotypal disorder  F22 – Delusional disorders  F23 – Brief psychotic disorder  F24 – Shared psychotic disorder  F25 – Schizoaffective disorders  F28 – Other psychotic disorder not due to a substance or known physiological condition  F29 – Unspecified psychosis not due to a substance or known physiological condition  F30 – Manic episode  F31 – Bipolar disorder  F32 – Major depressive disorder, single episode  F33 – Major depressive disorder, recurrent  F34 – Persistent mood (affective) disorders  F39 – Unspecified mood (affective) disorder  F40 – Phobic anxiety disorders  F41 – Other anxiety disorders  F42 – Obsessive-compulsive disorder  F43 – Reaction to severe stress, and adjustment disorders  F44 – Dissociative and conversion disorders  F44 – Dissociative amnesia  F45 – Somatoform disorders  F48 – Other nonpsychotic mental disorders  F51.0 – Insomnia not due to a substance or known physiological condition  G47.0 – Insomnia |
| Smell disturbance/anosmia | R43.0 – Anosmia  R43.1 – Parosmia |
| Taste disturbance/dysgeusia/ageusia | R43.2 – Parageusia |
| Thromboembolic disease | I26 – Pulmonary embolism  I80 – Superficial vein thrombosis  I80.1, I80.2, I81, I82.0, I82.2, I82.3, I82.4, I82.5 – Deep vein thrombosis |

ICD-10, *International Classification of Diseases, Tenth Revision*.

# Supplementary Table 6. Definitions for Covariates Included in the Weighting Model (Unless Otherwise Noted)

| **Covariate** | **Definition** |
| --- | --- |
| **Demographic characteristics**  *(measured at D0)* |  |
| Age | Continuous age |
| Sex | Male (ref)  Female  Unknown (missing) |
| Geographic region | Northeast (ref): Connecticut, Maine, Massachusetts, New Hampshire, New Jersey, New York, Pennsylvania, Rhode Island, Vermont  Midwest (North Central): Iowa, Illinois, Indiana, Kansas, Michigan, Minnesota, Missouri, North Dakota, Nebraska, Ohio, South Dakota, Wisconsin  South: Alabama, Arkansas, District of Columbia, Delaware, Florida, Georgia, Kentucky, Louisiana, Maryland, Mississippi, North Carolina, Oklahoma, South Carolina, Tennessee, Texas, Virginia, West Virginia  West: Alaska, Arizona, California, Colorado, Hawaii, Idaho, Montana, New Mexico, Nevada, Oregon, Utah, Washington, Wyoming  Other/missing/unknown: Armed Forces Americas, Armed Forces, Armed Forces Pacific, American Samoa, Micronesia, Guam, Marshall Islands, Northern Marianas Islands, Puerto Rico, Palau, Virgin Islands |
| Calendar time of hospitalization | Month/year |
| **Disease severity**  *(measured at D0 or D1)* |  |
| Oxygen support status | *Invasive mechanical ventilation/**ECMO* was the occurrence of any of the following procedures: invasive mechanical ventilation or ECMO  *High-flow oxygen/noninvasive ventilation* was the occurrence of any of the following procedures: noninvasive ventilation or high-flow oxygen  *Low-flow oxygen* was the occurrence of any of the following procedures: low-flow oxygen, any oxygen, excluding high-flow, noninvasive ventilation, invasive mechanical ventilation, or ECMO  *Room air* was the absence of the above procedures |
| ICU status | Yes or no |
| **Comorbidities**^7^  *(measured from D–365 to D–1, except where noted; diagnosis codes for Long COVID outcomes were not included in comorbidities)* |  |
| Infection (non–COVID-19) | Tuberculosis; septicemia; bacterial infections; fungal infections; HIV infection; hepatitis; viral infection; parasitic, other specified, and unspecified infections; sexually transmitted infections (excluding HIV and hepatitis); sequela of specified infectious disease conditions |
| Previous COVID infection  *(measured from D–365 to  D–-15)* | U07.1 |
| Blood | Nutritional anemia; hemolytic anemia; aplastic anemia; acute posthemorrhagic anemia; sickle cell trait/anemia; coagulation and hemorrhagic disorders; diseases of white blood cells; immunity disorders; postprocedural or postoperative complications of the spleen; other specified and unspecified hematologic conditions |
| Diabetes mellitus | Diabetes mellitus without complication; diabetes mellitus with complication; diabetes mellitus, type 1; diabetes mellitus, type 2; diabetes mellitus, due to underlying condition, drug- or chemical-induced, or other specified type |
| Metabolic | Malnutrition; disorders of lipid metabolism |
| Obesity | Obesity |
| Endocrine | Thyroid disorders; fluid and electrolyte disorders; cystic fibrosis; pituitary disorders; other specified and unspecified endocrine disorders |
| Digestive | Intestinal infection; esophageal disorders; gastroduodenal ulcer; gastrointestinal and biliary perforation; gastritis and duodenitis; other specified and unspecified disorders of stomach and duodenum; appendicitis and other appendiceal conditions; regional enteritis and ulcerative colitis; intestinal obstruction and ileus; diverticulosis and diverticulitis; anal and rectal conditions; peritonitis and intra-abdominal abscess; biliary tract disease; hepatic failure; other specified and unspecified liver disease; pancreatic disorders (excluding diabetes); gastrointestinal hemorrhage; noninfectious gastroenteritis; noninfectious hepatitis; postprocedural or postoperative digestive system complication |
| Neurologic | Meningitis; encephalitis; other specified CNS infection and poliomyelitis; Parkinson’s disease; multiple sclerosis; other specified hereditary and degenerative nervous system conditions; cerebral palsy; paralysis (other than cerebral palsy); epilepsy; convulsions; neurocognitive disorders; transient cerebral ischemia; coma; stupor; and brain damage; CNS abscess; polyneuropathies; myopathies; postprocedural or postoperative nervous system complication |
| Diseases of veins, lymphatic vessels, and lymph nodes, not elsewhere classified | Chronic phlebitis; thrombophlebitis and thromboembolism; varicose veins of lower extremity; post-thrombotic syndrome and venous insufficiency/hypertension; other specified diseases of veins and lymphatics |
| Diseases of arteries, arterioles, and capillaries | Peripheral and visceral vascular disease; arterial dissections; aortic, peripheral, and visceral artery aneurysms; aortic and peripheral arterial embolism or thrombosis |
| Diseases of pulmonary circulation | Acute pulmonary embolism; pulmonary heart disease |
| Ischemic heart diseases | Coronary atherosclerosis and other heart disease; complications of acute myocardial infarction; acute myocardial infarction |
| Cerebrovascular disease | Cerebral infarction; acute hemorrhagic cerebrovascular disease; sequelae of hemorrhagic cerebrovascular disease; occlusion or stenosis of precerebral or cerebral arteries without infarction; other and ill-defined cerebrovascular disease; sequelae of cerebral infarction and other cerebrovascular disease |
| Abnormal blood pressure (hyper) | Essential hypertension; hypertension with complications and secondary hypertension |
| Abnormal blood pressure (hypo) | Hypotension |
| Other and unspecified disorders of the circulatory system | Other specified and unspecified circulatory disease; acute phlebitis; thrombophlebitis and thromboembolism; vasculitis; postprocedural or postoperative circulatory system complication |
| Other forms of heart disease | Nonrheumatic and unspecified valve disorders; myocarditis and cardiomyopathy; pericarditis and pericardial disease; other and ill-defined heart disease |
| Heart failure | Heart failure |
| Conductive disorders, dysrhythmias | Conduction disorders; cardiac dysrhythmias; cardiac arrest and ventricular fibrillation |
| Rheumatic fever/diseases | Chronic rheumatic heart disease; acute rheumatic heart disease; endocarditis and endocardial disease |
| Renal (acute) | Nephritis, nephrosis, renal sclerosis; acute and unspecified renal failure |
| Renal (chronic) | Chronic kidney disease |
| Transplant | Complication of transplanted organs or tissue; organ transplant status |
| Genitourinary condition | Urinary tract infections; calculus of urinary tract; other specified and unspecified diseases of kidney and ureters; other specified and unspecified diseases of bladder and urethra; hematuria; proteinuria; vesicoureteral reflux; hyperplasia of prostate; inflammatory conditions of male genital organs; inflammatory diseases of female pelvic organs |
| Respiratory | Sinusitis; pneumonia (except that caused by tuberculosis); influenza; acute and chronic tonsillitis; acute bronchitis; other specified upper respiratory infections; other specified and unspecified upper respiratory disease; chronic obstructive pulmonary disease and bronchiectasis; asthma; aspiration pneumonitis; pleurisy, pleural effusion, and pulmonary collapse; respiratory failure, insufficiency, or arrest; lung disease due to external agents; pneumothorax; mediastinal disorders; other specified and unspecified lower respiratory disease; postprocedural or postoperative respiratory system complication |
| Mood/neuropsychiatric features | Schizophrenia spectrum and other psychotic disorders; depressive disorders; bipolar and related disorders; other specified and unspecified mood disorders; anxiety and fear-related disorders; obsessive-compulsive and related disorders; trauma- and stressor-related disorders; disruptive, impulse-control and conduct disorders; personality disorders; feeding and eating disorders; somatic disorders; suicidal ideation/attempt/intentional self-harm; miscellaneous mental and behavioral disorders/conditions; neurodevelopmental disorders |
| Behavioral | Alcohol-related disorders; opioid-related disorders; cannabis-related disorders; sedative-related disorders; stimulant-related disorders; hallucinogen-related disorders; inhalant-related disorders; other specified substance–related disorders; suicide attempt/intentional self-harm; subsequent encounter; opioid-related disorders, subsequent encounter; stimulant-related disorders, subsequent encounter; cannabis-related disorders, subsequent encounter; hallucinogen-related disorders, subsequent encounter; sedative-related disorders, subsequent encounter; inhalant-related disorders, subsequent encounter; mental and substance use disorders, sequela |
| Smoking | Tobacco-related disorders; history of smoking/tobacco use |
| Musculoskeletal/connective tissue | Infective arthritis; osteomyelitis; rheumatoid arthritis and related disease; juvenile arthritis; other specified chronic arthropathy; immune-mediated/reactive arthropathies; spondylopathies/spondyloarthropathy (including infective); systemic lupus erythematosus and connective tissue disorders; musculoskeletal abscess; aseptic necrosis and osteonecrosis; osteomalacia; autoinflammatory syndromes |
| Malformations | Cardiac and circulatory congenital anomalies; digestive congenital anomalies; genitourinary congenital anomalies; nervous system congenital anomalies; congenital malformations of eye, ear, face, and neck; cleft lip or palate; respiratory congenital malformations; musculoskeletal congenital conditions; chromosomal abnormalities; other specified and unspecified congenital anomalies |
| Factors influencing health status | Socioeconomic/psychosocial factors; lifestyle/life management factors |
| Neoplasms | Head and neck cancers – eye; head and neck cancers – lip and oral cavity; head and neck cancers – throat; head and neck cancers – salivary gland; head and neck cancers – nasopharyngeal; head and neck cancers – hypopharyngeal; head and neck cancers – pharyngeal; head and neck cancers – laryngeal; head and neck cancers – tonsils; head and neck cancers – all other types; cardiac cancers; gastrointestinal cancers – esophagus; gastrointestinal cancers – stomach; gastrointestinal cancers – small intestine; gastrointestinal cancers – colorectal; gastrointestinal cancers – anus; gastrointestinal cancers – liver; gastrointestinal cancers – bile duct; gastrointestinal cancers – gallbladder; gastrointestinal cancers – peritoneum; gastrointestinal cancers – all other types; respiratory cancers; bone cancer; sarcoma; skin cancers – melanoma; skin cancers – all other types; breast cancer – ductal carcinoma in situ; breast cancer – all other types; female reproductive system cancers – uterus; female reproductive system cancers – cervix; female reproductive system cancers – ovary; female reproductive system cancers – fallopian tube; female reproductive system cancers – endometrium; female reproductive system cancers – vulva; female reproductive system cancers – vagina; female reproductive system cancers – all other types; male reproductive system cancers – prostate; male reproductive system cancers – testis; male reproductive system cancers – penis; male reproductive system cancers – all other types; urinary system cancers – bladder; urinary system cancers – ureter and renal pelvis; urinary system cancers – kidney; urinary system cancers – urethra; urinary system cancers – all other types; nervous system cancers – brain; nervous system cancers – all other types; endocrine system cancers – thyroid; endocrine system cancers – pancreas; endocrine system cancers – thymus; endocrine system cancers – adrenocortical; endocrine system cancers – parathyroid; endocrine system cancers – pituitary gland; endocrine system cancers – all other types; Hodgkin lymphoma; non-Hodgkin lymphoma; leukemia – acute lymphoblastic leukemia; leukemia – acute myeloid leukemia; leukemia – chronic lymphocytic leukemia; leukemia – chronic myeloid leukemia; leukemia – hairy cell; leukemia – all other types; multiple myeloma; malignant neuroendocrine tumors; mesothelioma; myelodysplastic syndrome; cancer of other sites; secondary malignancies; malignant neoplasm, unspecified; neoplasms of unspecified nature or uncertain behavior; benign neoplasms; conditions due to neoplasm or the treatment of neoplasm |
| Other signs/symptoms | Syncope; shock; genitourinary signs and symptoms; circulatory signs and symptoms |
| **Baseline preinfection ICD-10 codes (symptoms consistent with Long COVID)**  *(measured from D–365 to D–15)* |  |
| Fatigue | Full code lists used for Long COVID outcomes are shown in Supplementary Table 5 |
| Dyspnea/breathlessness |  |
| Cough |  |
| Chest pain |  |
| Diarrhea |  |
| Dysautonomia |  |
| Smell disturbance/anosmia |  |
| Taste disturbance/dysgeusia/ageusia |  |
| Headache |  |
| Muscle pain/myalgia |  |
| Joint pain/arthralgia |  |
| Cerebrovascular disease |  |
| Cognitive dysfunction |  |
| Ischemic heart disease |  |
| Neuropsychiatric features |  |
| Thromboembolic disease |  |
| **Concomitant medications**  *(measured from D0 to D1)* |  |
| Corticosteroids^8^ | Hospital chargemaster data using “prednisone”, “prednisolone”, “methylprednisolone”, “hydrocortisone”, “dexamethasone”, “Decadron”, “Dexasone” , “Solurex”, “Baycadron”, “Deltasone”, “Orapred”, “Predicort”, “Prelone”, “Millipred”, “Medrol”, “Solu-Medrol”, “Hydrocort”, “Alphosyl”, “Aquacort”, “Cortef”, “Solu-Cortef”” as a text string  o*r*  Pharmacy claims and medical claims with generic names “cortisone acetate”, “prednisolone”, “methylprednisolone”, “dexamethasone”, “hydrocortisone”, “prednisone”  *or*  Procedures J1100, J1710, J1720, J2640, J2920, J2930, J7506, J7509, J7510, J7512, J8540, S0173 in medical claims and chargemaster data  Route of administration was limited to IV and oral |
| HIV protease inhibitors^9^ | Pharmacy claims using generic names “atazanavir”, “darunavir”, “fosamprenavir”, “indinavir”, “lopinavir”, “ritonavir”, “nelfinavir”, “saquinavir”, “tipranavir”, “nirmatrelvir”, “molnupiravir”  *or*  Pharmacy claims using brand names “Reyataz”, “Evotaz”, “Prezista”, “Prezcobix”, “Lexiva”, “Crixivan”, “Kaletra”, “Viracept”, “Norvir”, “Invirase”, “Aptivus”, “Paxlovid”, “Lagevrio”  *or*  Hospital chargemaster data using the brand and generic names above as a text string  *or*  Procedure S0140 in medical claims and chargemaster data |
| Immunomodulators^8^ | Included hospital chargemaster data or branded or generic medical or pharmacy claims for tocilizumab, sarilumab, baricitinib  *or*  Procedures C9264, J3262, XW033G5, XW033H5, XW043G5, XW043H5 in medical claims or chargemaster data |
| Convalescent plasma^10^ | Medical claims with procedures P9017, P9071, XW14325, P9099, XW13325, as well as hospital chargemaster data |
| Anticoagulant^*^ | Searches of hospital chargemaster data and pharmacy and medical claims for: “apixaban”, “argatroban”, “desirudin”, “lepirudin”, “dabigatran”, “danaparoid”, “edoxaban”, “tinzaparin”, “heparin”, “ardeparin”, “bivalirudin”, “Eliquis”, “Acova”, “Iprivask”, “Refludan”, “Pradaxa”, “Orgaran”, “Savaysa”, “Innohep”, “Normiflo”, “Angiomax”  *or*  Procedure codes J1644, J0883, C9121, J1945, Q2021, J1945, Q2021, C9107, J1655, C9111, J0583 in medical claims or chargemaster data  Codes/descriptions indicating use for lock flush, irrigation, dialysis, or assays were not included, nor were routes of administration of interest (intravenous or oral) |

CNS, central nervous system; D, day relative to hospital admission; ECMO, extracorporeal membrane oxygenation; ICD-10, *International Classification of Diseases, Tenth Revision*; ICU, intensive care unit; IV, intravenous.

^*^Gilead- and Aetion-defined.

# References

1. Agency for Healthcare Research and Quality, Healthcare Cost and Utilization Project. Clinical Classifications Software Refined (CCSR). Available at: [www.hcup-us.ahrq.gov/toolssoftware/ccsr/ccs_refined.jsp](http://www.hcup-us.ahrq.gov/toolssoftware/ccsr/ccs_refined.jsp). Accessed October 5, 2022.

2. Hernan MA, Robins JM. Causal Inference: What If. Boca Raton, FL: Chapman & Hall/CRC; 2020.

3.Soriano JB, Murthy S, Marshall JC, Relan P, Diaz JV, WHO Clinical Case Definition Working Group on Post-COVID-19 Condition. A clinical case definition of post-COVID-19 condition by a Delphi consensus. Lancet Infect Dis. 2022;22(4):e102-e7.

4. US Centers for Disease Control and Prevention. People who are immunocompromised 2023. Available at: <https://archive.cdc.gov/www_cdc_gov/coronavirus/2019-ncov/need-extra-precautions/people-who-are-immunocompromised.html>. Accessed December 10, 2024.

5. Patel M, Chen J, Kim S, Garg S, Flannery B, Haddadin Z et al. Analysis of MarketScan data from immunosuppressive conditions and hospitalizations for acute respiratory illness, United States. Emerg Infect Dis. 2020;26(8):1720-30.

6. Gulick RM, Pau AK, Daar E, Evans L, Gandhi RT, Tebas P, et al. National Institutes of Health COVID-19 Treatment Guidelines Panel: Perspectives and Lessons Learned. Ann Intern Med. 2024;177(11):1547-57. doi:10.7326/ANNALS-24-00464

7. Agency for Healthcare Research and Quality, Healthcare Cost and Utilization Project. Tools archive for Clinical Classifications Software Refined. Available at: <https://www.hcup-us.ahrq.gov/toolssoftware/ccsr/ccsr_archive.jsp>. Accessed August 14, 2023.

8. Barnado A, Casey C, Carroll RJ, Wheless L, Denny JC, Crofford LJ. Developing electronic health record algorithms that accurately identify patients with systemic lupus erythematosus. Arthritis Care Res (Hoboken). 2017;69(5):687-93.

9. Paul DW, Neely NB, Clement M, Riley I, Al-Hegelan M, Phelan M, et al. Development and validation of an electronic medical record (EMR)-based computed phenotype of HIV-1 infection. J Am Med Inform Assoc. 2018;25(2):150-­7.

10. Optum360. Coding for Coronavirus (COVID-19). Available at: <https://www.optum360coding.com/upload/docs/Optum360%20Coding%20for%20Coronavirus%20Final8.pdf>. Accessed August 14, 2023.
